# Indoor Large-Scale MIMO-Based RSSI Localization with Low-Complexity RFID Infrastructure

**DOI:** 10.3390/s20143933

**Published:** 2020-07-15

**Authors:** Mohammed El-Absi, Feng Zheng, Ashraf Abuelhaija, Ali Al-haj Abbas, Klaus Solbach, Thomas Kaiser

**Affiliations:** 1 Institute of Digital Signal Processing, University of Duisburg-Essen, 47057 Duisburg, Germany; feng.zheng@uni-due.de (F.Z.); ali.alhaj-abbas@uni-due.de (A.A.-h.A.); klaus.solbach@uni-due.de (K.S.); thomas.kaiser@uni-due.de (T.K.); 2 Electrical Engineering Department, Applied Science Private University, Amman 11931, Jordan; a_abualhijaa@asu.edu.jo

**Keywords:** localization, RFID, large-scale MIMO, passive RFID, RSSI, localization accuracy

## Abstract

Indoor localization based on unsynchronized, low-complexity, passive radio frequency identification (RFID) using the received signal strength indicator (RSSI) has a wide potential for a variety of internet of things (IoTs) applications due to their energy-harvesting capabilities and low complexity. However, conventional RSSI-based algorithms present inaccurate ranging, especially in indoor environments, mainly because of the multipath randomness effect. In this work, we propose RSSI-based localization with low-complexity, passive RFID infrastructure utilizing the potential benefits of large-scale MIMO technology operated in the millimeter-wave band, which offers channel hardening, in order to alleviate the effect of small-scale fading. Particularly, by investigating an indoor environment equipped with extremely simple dielectric resonator (DR) tags, we propose an efficient localization algorithm that enables a smart object equipped with large-scale MIMO exploiting the RSSI measurements obtained from the reference DR tags in order to improve the localization accuracy. In this context, we also derive Cramer–Rao lower bound of the proposed technique. Numerical results evidence the effectiveness of the proposed algorithms considering various arbitrary network topologies, and results are compared with an existing algorithm, where the proposed algorithms not only produce higher localization accuracy but also achieve a greater robustness against inaccuracies in channel modeling.

## 1. Introduction

Recently, passive radio frequency identification (RFID) has been identified as enabling technology for many applications, such as localization, tracking, and internet of things (IoTs), due to an extreme reduction of costs, low energy consumption, and low complexity [1,2,3,4,5]. The global positioning system (GPS) was an immense step towards ubiquitous localization, where it mostly provides an acceptable level of accuracy for outdoor applications [4]. Unfortunately, GPS technology does not work appropriately in indoor environments since the satellite signal is attenuated critically, and line-of-sight (LOS) links between the node and satellites are required. Moreover, GPS requires expensive infrastructure, whereas future technologies necessitate efficient energy consumption and cost reduction. In this regard, passive RFID-based localization addresses these issues and plays an effective role to perform localization for the future wireless applications.

RFID technology enables a reader to interrogate and communicate with tags through the backscattered signal. The RFID system is broadly classified, according to the tag type, into active, semi-passive, and passive tags. This classification mainly depends upon the existence and reliance on a battery source in the tag. An active or semi-passive tag embeds an internal battery, where the battery continuously powers the active tags and its radio-frequency (RF) communication circuitry. Passive tags do not contain a battery and totally depend on the power harvested from the reader’s signal. The passive tags are generally smaller in size and cheaper than the active tags, providing a promising solution to a wide range of applications.

Recently, the operation of RFID systems has been proposed to be moved from UHF and ultrawide band (UWB) into the millimeter-wave (mm-wave) band in order to overcome the limitations at UHF and UWB bands such as bandwidth limitations and power regulations, respectively [6]. Furthermore, mm-wave RFID systems enable the equipment of large-scale MIMO antennas at the reader and tag sides with practical dimensions [7]. Accordingly, with large-scale MIMO, a large available bandwidth and a high transmit power allowance, the mm-wave technology is considered as a potential candidate for RFID based localization systems.

To estimate an object location, two or more anchor nodes with known positions should exist in the coverage of the object in order to determine the location of the object via noisy ranging techniques such as Received Signal Strength Indicator (RSSI) [8], Time of Arrival (ToA) [2,9], Angle of Arrival (AoA) [10], etc. Among these ranging techniques, RSSI is considered in this work, since it meets future application requirements including the hardware simplicity and low cost [11,12]. However, RSSI ranging suffers from imprecise accuracy due to the various environment factors such as multipath, especially in indoor environments [13,14,15]. Accordingly, improving RSSI localization is worth studying to better exploit its advantages in future applications [16,17,18,19].

Generous research effort has been devoted to developing localization methods that can compensate for the adverse effects of uncertainties in RSSI measurements [16,17,18,19,20,21,22,23,24,25,26,27,28,29]. Most of these proposed methods depend on the optimization techniques or fingerprinting, which requires high computational complexity, making them unpractical for IoT localization applications. Moreover, to the best of our knowledge, only the works in [30,31] consider large-scale, MIMO-based mm-wave RFID localization. The authors of [30] investigate the possibility to localize passive tags by adopting a single mm-wave RFID reader performing beamsteering, in which an accuracy of several centimeters is achieved using AoA ranging. RSSI localization at mm-wave RFID system is considered in [31] obtaining meter-level positioning accuracy. Recently, we propose in [2] a highly accurate indoor localization based on RFID systems using time-based ranging at terahertz band and evaluate it in [32] using a testbed at low GHz band. However, this approach does not offer the low-complexity that is needed for IoT applications.

Motivated by IoT applications that need accurate and simple localization algorithms with simple infrastructure, we consider in this paper to develop an RSSI localization algorithm in order to increase the localization accuracy with moderate computational complexity. Thanks to the channel hardening of the large-scale MIMO technology that is promised at mm-wave band, making the small-scale quickly diminish with the increase of the array size [33,34], a large-scale MIMO setup at the mm-wave reader is proposed in this work as a promising candidate for RSSI localization in order to increase the localization accuracy.

Inspired by large-scale MIMO, which has become a reality at mm-wave band, and the extremely simple localization infrastructure, which should be as simple as possible in order to minimize installation cost, our key contributions in this work are summarized in the following.

1. We propose large-scale MIMO-based RSSI localization at mm-wave band using the extremely simple dielectric resonator (DR) tags as the anchor node infrastructure in order to improve the self-localization accuracy of smart objects. Unlike UHF and UWB, mm-wave band offers the advantage of equipping large-scale MIMO structure at the reader and tag sides. The reader is equipped with a large-scale antenna in order to benefit from the channel hardening, making the small-scale quickly diminish with the increase of the array size. Furthermore, each reference DR tag is designed to have its unique resonance frequency and to have an array of DR elements in order to extend the ranging coverage. The validation of the DR tag is presented using measurements.
2. We apply the weighted linear least-squares (WLLS) estimator combined with the optimal large-scale MIMO-based ranging technique in order to estimate the position of the object.
3. We derive the Cramér–Rao Lower Bound (CRLB) for the variance of the position estimator for the proposed framework.
4. Two sub-optimal algorithms are proposed, which can approach the performance of the optimal large-scale MIMO-based ranging with low computational complexity.
5. Simulations are performed using analytical and deterministic channels to demonstrate the performance of the proposed algorithms considering various topologies of the infrastructure. These results show that the proposed method significantly improves the localization accuracy with simple hardware and computational complexity.

This paper is organized as follows. We give a discussion of related work in Section 2. The system model is described in Section 3. We present in Section 4 the proposed large-scale MIMO-based RSSI localization. Section 5 presents the proposed sub-optimal algorithms. Measurement and simulation setup is presented in Section 6. Section 7 reports simulative results. We conclude our work in Section 8.

## 2. Related Work

Several studies have been conducted in the literature to investigate RFID-based localization using the RSSI technique in order to improve its accuracy. Accordingly, comprehensive channel models for the RFID channel are proposed and utilized to enhance RSSI technique accuracy, as in [16,20,21,35,36] and references therein. The channel model describing the relation between RSSI and distance is found experimentally in [20] for a passive RFID system. This channel model aims at improving the ranging accuracy, and hence the localization accuracy. The effect of carrier frequency on the channel model and localization accuracy is investigated in [21], where the considered model explicitly depends on the transmit power and the transmission frequency. The authors of [16] consider a 3D antenna radiation pattern in the channel model, where the accuracy of RSSI-based localization is investigated and the CRLB is derived.

Another direction to enhance RSSI localization is to use pattern matching and fingerprinting based methods for reducing the influence of range measurement errors [17,19,22,23,24,25,26,27,28,29,37,38,39,40,41]. The LANDMARC indoor localization system is presented in [22] as a pattern matching method to enhance the overall accuracy of locating objects using some reference tags. In order to improve the LANDMARC algorithm, the work in [37] proposes the weighted path length and support vector regression algorithm, while the work in [38] proposes Bayesian probability and k-nearest neighbor based indoor positioning algorithms. In [23], the LANDMARC approach is improved to overcome the limitations of the multipath effects and RF interference. In the Multidimensional Scaling (MDS) localization technique introduced in [24], another pattern matching method is proposed to improve a fingerprinting localization algorithm. The MDS-based localization algorithm is extended in [39] to mitigate the multipath environment toward improving localization accuracy for passive RFID localization. Recently, the authors of [19] combined the fingerprinting localization technology and the nonmetric MDS algorithm to achieve high accuracies in the RFID multi-tag cooperative localization method in the indoor environment. In [17], a similarity analysis approach with reference tags is proposed for improving the accuracy of range-based localization algorithms using a grid-based pattern with many reference tags. The idea behind is to divide the tags into groups according to transmission distances, and all tags in the same group have the same similarity in propagation distances. The similarity measure is calculated from the combination between RSSI and signal phases. The authors of [25] consider the problem of self-localization by a resource-constrained node given perturbed anchor position information and distance estimates from the anchor nodes, where a closed-form, weighted, least-squares solution is proposed. The work in [40] proposes a Gaussian filtering algorithm based on an extreme learning machine to address the problem of inaccurate indoor positioning when significant RSSI fluctuations happen during the measurement process. In [26], continuous wavelet transform is used to extract time-frequency or time-scaling information from each RSSI samples for indoor fingerprinting localization algorithm. The work in [27] proposes to use the feature-scaling-based k-nearest neighbor algorithm with RSSI. In [28,29], neural network and machine learning algorithms are used for RSSI fingerprints, respectively, in order to improve the localization accuracy. Even fingerprint methods offer higher accuracy and better robustness, they require more cost in the facility and more complexity, which does not meet IoT expectations [4]. The work in [41] proposes to use mid-grained intermediate-level channel measurements that are provided by the IEEE 802.11ad/ay standards to construct the fingerprinting database.

Large-scale MIMO technology is also proposed as an attractive candidate for localization aiming at improving estimation accuracy in 5G cellular communication systems using mostly AoA ranging techniques as in [42,43,44,45,46,47] and references therein. For instance, the authors of [43] propose a finger-printing localization solution for locating multiple users by means of distributed massive MIMO. The work in [44] proposes a personal mobile radar with mm-wave massive arrays, which is used for localization and mapping as in [45]. A direct localization approach is proposed in [46], where the position of a user is localized by jointly processing the observations obtained at distributed massive MIMO base stations. In [47], an architecture with distributed antenna units with a multistage/multiresolution search strategy is proposed in line-of-sight indoor environments. It is worth to highlight that the considered works in the literature under large-scale MIMO need more complex hardware and infrastructure requirements in order to find the AoA and ToA measurements, where this is not the case in our new proposed system.

## 3. System Model

**Figure 1:**
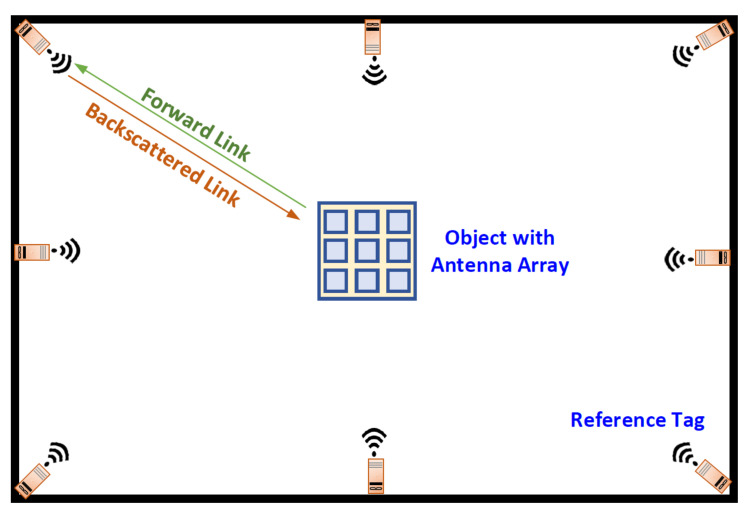
2D radio frequency identification (RFID) localization system with monostatic configuration.

A monostatic mm-wave RFID-based localization system is considered, where a number of retro-reflective tags operating at mm-wave frequencies with known geographic positions are nested in the room acting as reference tags as shown in Figure 1. An object, i.e., a reader, not aware of its position broadcasts an RF signal at a fundamental frequency and collects the backscatter signals from the surrounding tags in order to measure the RSSI backscattered from the retro-reflective tags. Afterwards, from the RSSI measurements from the reference tags, the distance between the reader and the tags is estimated, and then the position of the reader is estimated using the positioning algorithms [48].

### 3.1. Tag Setup

**Figure 2:**
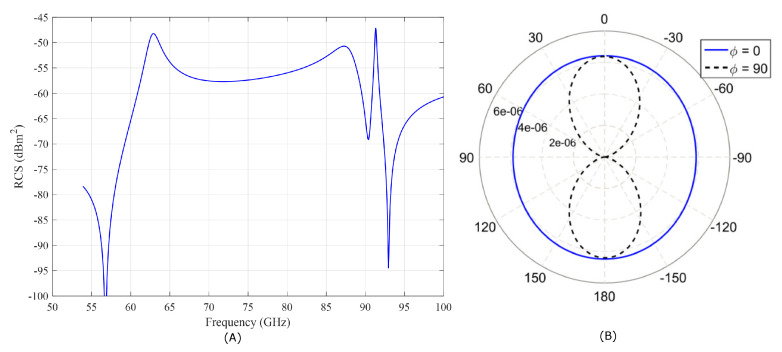
(**A**) The monostatic radar cross-section (RCS) for a single dielectric resonator (DR) tag. (**B**) The bi-static scattering cross-section ($m^{2}$) for a plane wave incident along the z-axis at 60.24 GHz (resonance frequency) on the plane ϕ = 0 and π/2.

Recently, one of the simplest passive tags has been proposed using dielectric resonators (DRs) in [49], which has neither battery nor chip on it. Based on this, the DR tags in this work are used as reference anchors placed in the localization environment. The DR tag proposed here operates as a DR antenna that receives an incident electromagnetic wave which hits the dielectric body and fully re-transmits the received power by generating a retro-directive reflected wave, which the reader can detect. By exciting the dielectric resonator at one of its resonant frequencies, a resonance field mode is induced by the incident wave. Compared to the scattered field amplitudes outside the resonance frequency, the reflection from the DR tag is much stronger at the resonance frequency [3,49,50,51]. The retro-directive scattering of the incident plane wave by the DR tag can be described by the monostatic radar cross-section (RCS), which is frequency dependent (peak at the resonance frequencies) and depends on the DR material (relative permittivity), size, and the excited resonant mode. For a realistic modeling of the behavior of our DR tag, we constructed 3-D models in a EM-simulator (CST Microwave Studio) [52] to perform full-wave electromagnetic simulation. A single ceramic cylindrical resonator with relative permittivity of 35, radius of 0.682 mm, and height of 0.64 mm was simulated, where and Figure 2A shows the calculated monostatic RCS spectral signature with RCS values of −47.18 and −43.28 ${\mathrm{dBm}}^{2}$ at resonance peaks at around 58 and 60.24 GHz, respectively, and Figure 2B shows the bi-static scattering cross-section of the simulated tag. We see that these RCS values are quite low, which is a serious problem concerning the detection of the tag by a reader in several meters distance.

Due to the capability of fabricating a large-scale DR array at mm-wave band in a very small area, we propose to use a massive DR array aiming at increasing the RCS of the chipless tag to overcome the low scattering response of the DR seen by the reader. Accordingly, the DR array at the tag is proposed to be planar array with $K=K_{1}K_{2}$ DR elements placed in the XY plane, where $K_{1}$ DR elements are placed in each row along the *x*-axis direction and $K_{2}$ DR elements are placed in each column along the y-axis direction with λ/2 spacing between the DR elements.

**Figure 3:**
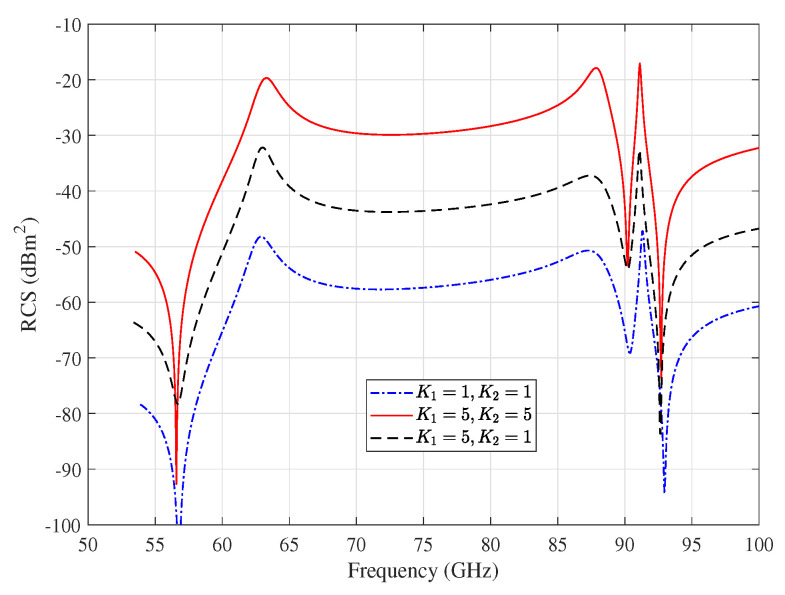
Monostatic RCS of DR array for different planar DR array configurations.

Figure 3 shows the monostatic RCS spectrum of the array for the cases $K_{1}=5,K_{2}=1$ and $K_{1}=K_{2}=5$. Approximately, 14 dB and 28 dB improvement in the RCS at the resonance frequencies for the cases of $K_{1}=5,K_{2}=1$ and $K_{1}=K_{2}=5$ are achieved, respectively. In the meantime, the improvement in monostatic RCS of massive planar DR arrays can be estimated, as shown in Figure 3, to be $10\log{\left(K_{1}K_{2}\right)}^{2}$ dB. For example, an array area of 11 × 11 mm with 5×5 elements can improve the RCS by 28 dB. In other words, increasing the RCS of the tag improves its receiving and reflecting gain, which enhances the detectability of the tag.

The reference DR tags can be frequency coded in a way that each tag has its unique resonance frequency with high RCS, whereas the other tags have low RCS at this operating frequency. The resonance frequency of the tags can be designed by controlling the DR size, dielectric constant, and excited mode. As an example, assume two DR tags with 5×5 array of elements: the first is designed to have its first resonance frequency at 63 GHz, as shown in Figure 3, while the second is designed to have it at 70 GHz. When the reader communicates with the first tag at its resonance frequency 63 GHz, the first tag is excited at one of its resonant frequencies and reflects a peak much higher than the reflected signal from the second, as shown from Figure 3. Accordingly, the inter-tag interference can be neglected with a precise design for the reference tags. Due to the large bandwidth at mm-wave band, the DR tag design in terms of coding capacity is possible for many chipless DR tags as in [53,54,55] and references therein.

In the case of localizing multiple readers in the same environment, the retro-directivity of the designed tag reduces the interference of RSSI measurements, where the interference can occur when the objects are close to each other. This can be solved by increasing the number of the reference tags in the environment in order to decrease the probability of the objects to be connected to the same reference tags.

### 3.2. System Configuration

We consider a two-dimensional indoor scenario with dimension $w_{1}\times w_{2}$, in which *N* DR chipless tags with different resonant frequencies are composed in order to help a reader equipped with a planar array of *M* identical antennas to localize itself. Here, only 2D localization is considered, but extension to the 3D case is straightforward. Let $p_{n}={\left[x_{n},y_{n},f_{n}\right]}^{T}\in R^{3},\;n\in \left\{1,2,\ldots ,N\right\}$ be the known settings of the *n*th tag, where $x_{n}$ and $y_{n}$ are x- and y-coordinates of the tag, respectively; and $f_{n}$ is the resonance frequency of the tag. A basic requirement for the distribution of the tags is as follows: these reference tags should be distributed in the environment in order to guarantee that the object is always covered by at least three reference tags.

Denote by $p_{r}={\left[\bar{x},\bar{y}\right]}^{T}\in R^{2}$ the reader coordinate vector to be estimated, which refers also to the antenna array centroid, and $p_{r}^{m}={\left[{\bar{x}}_{m},{\bar{y}}_{m}\right]}^{T}\in R^{2}$ is the coordinate vector of the *m*th antenna.

### 3.3. RSSI Modeling

During the localization process and in order to estimate the distance between the reader and the *n*th tag, the backscattered RSSI from the *n*th tag is measured through two modes. In the transmitting mode, the reader performs an exhaustive search based on finding the optimal signal-to-noise ratio (SNR) in order to discover the *n*th tag by probing in all directions of the reader. At the receiving mode, if the frequency of the incident signal matches the resonant frequency of the tag located within the reader beam, the tag will backscatter RF power towards the reader. On the other hand, if the frequency of the incident signal does not match the resonant frequency, the tag response will be very weak compared to the resonance case. At the reader side and contrary to the transmitting mode, no beamforming is required at the receiving mode, and, hence, the reader has no favored receiving direction. Moreover, the received signals across the array elements are not combined. In addition to the tag’s backscatter signals, the reader also receives undesired reflections from the environment.

As the wavelengths of mm-wave radio signals are extremely short, the distances between the antennas in the array can be made very short, i.e., multiple of wavelengths. As a result, the distance between the *n*th tag and the centroid of the reader $d_{n}$ can be approximated using small-angle approximations to be the distance between the *n*th tag at the *m*th antenna $d_{mn}$. Accordingly, the total received power backscattered from the *n*th tag at the *m*th antenna on the reader at frequency $f_{o}$ with $\lambda_{o}$ wavelength is [16,56]

(1) $${\bar{P}}_{mn}^{f_{o}}=\frac{4\pi }{\lambda_{o}^{2}}\sigma_{n}\rho_{n}^{2}P_{T}G_{R}^{f_{o}}{\bar{G}}_{R}^{f_{o}}{\left|\bar{L}\left(d_{mn},f_{o}\right)h_{n}h_{mn}\right|}^{2},$$

where $\sigma_{n}$ is RCS of the *n*th tag, and $\rho_{n}$ is the polarization loss factor due to the mismatch between the polarization of a reader antenna and the *n*th tag antenna. $P_{T}$ is the power transmitted by the RFID reader, which has total array gain of $G_{R}^{f_{o}}$ at frequency $f_{o}$ (at transmitting mode), while the single element antenna gain is ${\bar{G}}_{R}^{f_{o}}$ at frequency $f_{o}$ (at receiving mode). The term $\bar{L}\left(d_{mn},f_{o}\right)$ is channel path loss in the free-space propagation environment which is given by

(2) $$\bar{L}\left(d_{mn},f_{o}\right)={\left(\frac{\lambda_{o}}{4\pi d_{mn}}\right)}^{2}.$$

The symbol $h_{n}$ is the link fading coefficient between the *n*th tag and the reader, while $h_{mn}$ is the link fading coefficient between the *n*th tag and the *m*th antenna on the reader. Multipath fading is generally a challenge for wireless systems, and especially poses great problems for localization systems [39,57]. Accordingly, many researchers investigated the modeling of $h_{mn}$ to be characterized by log-normal, Rician, or Rayleigh distributions [16,56,57,58]. In this work, we used the log-normal path loss model, which is also extensively used in indoor environments to model the signal attenuation between DR reference tags and the reader in different frequency bands [16,19,58,59,60,61,62,63,64,65,66,67].

Specifically, the work in [64] shows the efficiency of the log-normal path loss model, i.e., also called close-in free space reference distance (CI), at mm-wave band, which provides simple calculations of large-scale path loss at different environments and frequency bands. This model is described as follows:

(3) $$\begin{array}{cc} L\left(d_{mn},f\right)\left[\mathrm{dB}\right] & =\bar{L}\left(d_{o},f_{o}\right)\left[\mathrm{dB}\right]-10\alpha \log_{10}\left(\frac{d_{mn}}{d_{o}}\right)\\ \; & -10\beta \log_{10}\left(\frac{f}{f_{o}}\right)+X_{mn}, \end{array}$$

where $L(d_{mn},f)$ is the average large-scale path loss at frequency *f* and distance $d_{mn}$, and $d_{o}$ is a close-in reference distance (usually 1 m). α and β are the path loss and frequency exponent, respectively, and $X_{mn}∼N\left(0,\sigma^{2}\right)$ (in dB) models the shadowing effect. For simplicity and without loss of generality, we assume that $\left\{X_{mn}\right\}$ are zero-mean uncorrelated (for different *m* and *n*) Gaussian processes with known and equal variances $\sigma^{2}$.

It is worth mentioning here that the effect of the surrounding environment is characterized using α and $X_{mn}$ in the log-normal path loss model, where many researchers have reported values for those parameters depending of measurements as in [63,64,65,66,67] and references therein.

Accordingly, the total backscattered received power at frequency *f* in (1) can be described using the log-normal path loss model as [60]

(4) $${\bar{P}}_{mn}^{f}\left[\mathrm{dBm}\right]={\bar{P}}_{d_{o}n}^{f}\left[\mathrm{dBm}\right]-20\alpha \log_{10}\left(\frac{d_{mn}}{d_{o}}\right)+X_{mn},$$

where ${\bar{P}}_{d_{o}n}^{f}$ is the received power at distance $d_{o}$ from the *n*th tag, which collects all constant factors in the propagation model in (1). Next, $d_{o}$ is assumed to equal 1 m.

## 4. Large-Scale MIMO-Based RSSI Localization

Unfortunately, the multipath propagation causes fast fluctuations of the reader’s received power at a given position due to constructive and destructive superposition of multipath signals, which could result in larger distance estimation errors for more distant tags compared to closer ones. The large number of antennas at the reader is utilized to increase the accuracy of RSSI localization. It has been shown that large-scale MIMO technology can overcome the effect of small-scale fast fading on system performance [33,34].

In this context, we propose large-scale MIMO-based RSSI localization using the backscattered signals from the DR tags. As the reader is equipped with large-scale MIMO, the received signal at each antenna is measured in order improve RSSI localization by benefiting from the channel hardening making the small-scale quickly fade to diminish with the increase in array size. The algorithm starts from finding the location of each antenna on the reader through two steps [14,15]. The first step is ranging, in which each antenna on the reader measures the RSSI from all the available tags and estimates the distances between this antennas and the tags as will be discussed in Section 4.1. The second step is called lateration or position estimation, in which the position of each antenna is estimated using the distances obtained from the ranging step by means of the trilateration algorithm, as will be discussed in Section 4.2 [2,14]. Section 4.3 derives CRLB of the localization error for the proposed algorithm.

### 4.1. Ranging

In the localization process, to perform ranging between the *m*th antenna on the reader and the *n*th tag, the *m*th antenna on the reader broadcasts a signal with the resonance frequency of the *n*th tag, i.e., $f_{n}$. Accordingly, the reader receives three types of signals. The first is the backscattered signal form the desired tag as discussed in Section 3.1, while the second is the inter-tag interference resulting from the backscattered signals from the other DR tags, where these signals are highly attenuated and degraded as mentioned in Section 3.1. The environment response is the third type, which is modeled using the log-normal channel model as argued in the previous section. In this work, inter-tag interference is neglected since it is very weak compared to the environment response.

Thus, the ranging between the *m*th antenna and the DR tags requires broadcasting *N* signals from the *m*th antenna with the different resonant frequencies, which are related to the resonant frequencies of the DR tags, within different time slots. It is assumed that the time slots are separated far enough from each other to avoid interference from other transmissions in other time slots. Without loss of generality and assuming that all tags are in the reader’s interrogation zone, the *m*th antenna frontend receives the backscattered signals from the DR tags and obtains the RSSI measurements. The RSSI vector of the *m*th antenna on the reader after ranging all DR tags is defined as

(5) $$r_{m}=\left({\bar{P}}_{m1}^{f_{1}},{\bar{P}}_{m2}^{f_{2}},..,{\bar{P}}_{mN}^{f_{N}}\right).$$

Afterwards, the distance vector is evaluated from $r_{m}$ using (4) as [68]

(6) $${\tilde{d}}_{m}=\left({\tilde{d}}_{m1},{\tilde{d}}_{m2},..,{\tilde{d}}_{mN}\right),$$

where ${\tilde{d}}_{mn}$ is the noise-polluted distance for $d_{mn}$ and expressed as ${\tilde{d}}_{mn}=d_{mn}10^{\frac{X_{mn}}{20\alpha }}$.

Accordingly, those estimated distances follow a log-normal random distribution expressed as

(7) $$\begin{array}{cc} {\tilde{d}}_{mn} & ∼10^{N\left(\log_{10}\left(d_{mn}\right),\frac{\sigma^{2}}{{\left(20\alpha \right)}^{2}}\right)}\\ \; & ∼e^{N\left(\mu_{mn},\sigma_{s}^{2}\right)} \end{array}$$

where $\mu_{mn}=\log\left(d_{mn}\right)$ is the mean of the normal distribution of $\log\left({\tilde{d}}_{mn}\right)$, and its standard deviation is expressed as $\sigma_{s}=\frac{\sigma \log10}{20\alpha }$. In the following, we use the notation ${\tilde{d}}_{mn}$ ∼ $\mathrm{LN}(\mu_{mn},\sigma_{s}^{2})$ to refer that $\log\left({\tilde{d}}_{mn}\right)$ is normally distributed with mean $\mu_{mn}$ and variance $\sigma_{s}^{2}$.

Note that the *k*th moment of a log-normal random variable of parameters $\left(\mu_{mn},\sigma_{s}\right)$ is given by [69]

(8) $$E\left[{\tilde{d}}_{mn}^{k}\right]=e^{\left(k\mu_{mn}+\frac{k^{2}\sigma_{s}^{2}}{2}\right)}.$$

Therefore, the mean of ${\tilde{d}}_{mn}$ is expressed as

(9) $$E\left[{\tilde{d}}_{mn}\right]=d_{mn}e^{\frac{\sigma_{s}^{2}}{2}},$$

and its variance is

(10) $$\mathrm{var}\left({\tilde{d}}_{mn}\right)=E\left[{{\tilde{d}}_{mn}}^{2}\right]-{\left(E\left[{\tilde{d}}_{mn}\right]\right)}^{2}=d_{mn}^{2}\left(e^{2\sigma_{s}^{2}}-e^{\sigma_{s}^{2}}\right).$$

### 4.2. Location Estimation Methods

In literature, there exist many location estimation methods to improve the localization accuracy, where they differ in the achieved estimation accuracy and computational complexity. The maximum-likelihood (ML) method achieves the optimal performance with high computational complexity [8,70]. To reduce the computational complexity of the ML method, it has been relaxed to a convex optimization problem in the form of a semidefinite program as in [71]. A less complex algorithm is non-linear least-squares estimation (Non-LSE) as discussed in [72]. Another approach is to reformulate the nonlinear equations constructed from the noisy RSSI measurements to some linear forms such as linear least-squares (LLS), as shown in [12,59] and references therein.

It is worth highlighting that the accuracy of the location estimation methods is highly dependent on the ranging accuracy. Therefore, improving ranging accuracy is vital in order to improve the localization accuracy. In this work, we developed large-scale MIMO-based RSSI localization framework, which can be applied to the aforementioned estimation methods. However, due to space limitations, we consider in this work only the WLLS estimator. Note that it is straightforward to extend the approach to other estimation algorithms.

In WLLS estimation, the nonlinear relations between the noisy distance vector ${\tilde{d}}_{m}$ and the unknown position of the *m*th antenna $p_{r}^{m}$ are described by [59,73]

(11) $${\tilde{d}}_{mn}^{2}={\left(x_{n}-{\bar{x}}_{m}\right)}^{2}+{\left(y_{n}-{\bar{y}}_{m}\right)}^{2},\;\;n\in \left\{1,2,\ldots ,N\right\}.$$

After mathematical manipulation, the nonlinear equations are formulated to be in a linear form as follows [59,73]

(12) $$H\;.\;p_{r}^{m}={\tilde{z}}_{m},$$

where

(13) $$H=\left[\begin{array}{cc} 2(x_{2}-x_{1}) & 2(y_{2}-y_{1})\\ \vdots & \vdots \\ 2(x_{N}-x_{1}) & 2(y_{N}-y_{1}) \end{array}\right],$$

and

(14) $${\tilde{z}}_{m}=\left[\begin{array}{c} x_{2}^{2}-x_{1}^{2}+y_{2}^{2}-y_{1}^{2}+{\tilde{d}}_{m1}^{2}-{\tilde{d}}_{m2}^{2}\\ \vdots \\ x_{N}^{2}-x_{1}^{2}+y_{N}^{2}-y_{1}^{2}+{\tilde{d}}_{m1}^{2}-{\tilde{d}}_{mN}^{2} \end{array}\right].$$

The model in (12) can be solved using WLLS estimator to estimate the position of the reader as follows [73]

(15) $${\hat{p}}_{r}^{m}={\left(H^{T}W_{m}H\right)}^{-1}H^{T}W_{m}{\tilde{z}}_{m},$$

where $W_{m}$ is the weighting matrix calculated as the inverse of the covariance matrix of the vector ${\tilde{z}}_{m}$. With the assumption that the distance estimations are independent, the covariance matrix of $z_{m}$ can be easily obtained as

(16) $$Q_{m}=\left[\begin{array}{cccc} \mathrm{var}\left({{\tilde{d}}_{m1}}^{2}\right)+\mathrm{var}\left({{\tilde{d}}_{m2}}^{2}\right) & \mathrm{var}\left({\tilde{d}}_{m1}^{2}\right) & \cdots & \mathrm{var}\left({{\tilde{d}}_{m1}}^{2}\right)\\ \mathrm{var}\left({{\tilde{d}}_{m1}}^{2}\right) & \mathrm{var}\left({{\tilde{d}}_{m1}}^{2}\right)+\mathrm{var}\left({{\tilde{d}}_{m3}}^{2}\right) & \cdots & \mathrm{var}\left({{\tilde{d}}_{m1}}^{2}\right)\\ \vdots & \vdots & \ddots & \vdots \\ \mathrm{var}\left({{\tilde{d}}_{m1}}^{2}\right) & \mathrm{var}\left({{\tilde{d}}_{m1}}^{2}\right) & \cdots & \mathrm{var}\left({{\tilde{d}}_{m1}}^{2}\right)+\mathrm{var}\left({{\tilde{d}}_{mN}}^{2}\right) \end{array}\right]$$

where var(.) denotes the variance of the term inside the brackets. Accordingly

(17) $$\mathrm{var}\left({{\tilde{d}}_{mn}}^{2}\right)=E\left[{{\tilde{d}}_{mn}}^{4}\right]-{\left(E\left[{{\tilde{d}}_{mn}}^{2}\right]\right)}^{2}.$$

Since ${\tilde{d}}_{mn}$ follows a log-normal random distribution as in (7), it can be derived that

(18) $$\mathrm{var}\left({\tilde{d}}_{mn}^{2}\right)=d_{mn}^{4}\left(e^{8\sigma_{s}^{2}}-e^{4\sigma_{s}^{2}}\right).$$

In a real application, it is mandatory to approximate the real distance $d_{mn}$ by the estimated distance ${\tilde{d}}_{mn}$.

As a result, the geometric position of the *m*th antenna ${\hat{p}}_{r}^{m}$ is estimated using WLLS. These steps are performed for all antennas on the reader, yielding the position estimation vectors ${\hat{p}}_{r}^{1},{\hat{p}}_{r}^{2},..,{\hat{p}}_{r}^{M}$ of all the antennas. Finally, the position of the object ${\hat{p}}_{r}$ is determined to be the centroid of the estimated antenna positions.

### 4.3. Cramér–Rao Lower Bound Derivation

In this part, we derive the CRLB of the localization error for the proposed algorithm. CRLB sets a lower bound for the variance of any unbiased estimator [8,12]. In this algorithm, the exact location of the object (reader) is estimated using the measurements obtained at the different antennas from the *N* tags. Accordingly, the observation vector is MN-dimensional RSSI collected independently, where the joint probability density function (PDF) of these RSSI measurements using (4) can be written as [8]

(19) $$\begin{array}{c} f_{p_{r}}\left({\bar{P}}_{mn}^{f}\right)=\prod_{m=1}^{M}\prod_{n=1}^{N}\frac{10}{\log10\sqrt{2\pi \sigma^{2}}{\bar{P}}_{mn}^{f_{n}}}\times \exp\left(-\frac{b}{8}{\left(\log\frac{d_{mn}^{2}}{{\hat{d}}_{mn}^{2}}\right)}^{2}\right), \end{array}$$

where $b={\left(\frac{20\alpha }{\sigma \log10}\right)}^{2}$ and ${\hat{d}}_{mn}={\left(\frac{{\bar{P}}_{d_{o}n}^{f_{n}}}{{\bar{P}}_{mn}^{f_{n}}}\right)}^{\frac{1}{2\alpha }}$.

**Theorem** **1.** *The CRLB of an unbiased position estimator ${\hat{p}}_{r}$ based on the proposed algorithm and the PDF in (19) is given by*

(20) $$var\left({\hat{p}}_{r}\right)=\frac{\sum_{m=1}^{M}\sum_{n=1}^{N}\frac{1}{d_{mn}^{2}}}{b\sum_{m=1}^{M}\sum_{n=1}^{N}\sum_{i=1}^{M}\sum_{k=1}^{N}Y_{n,m,i,k}},$$

*where*

$$\begin{array}{c} Y_{n,m,i,k}=\frac{\cos\phi_{mn}\sin\phi_{ik}\sin\left(\phi_{ik}-\phi_{mn}\right)}{d_{mn}^{2}d_{ik}^{2}}. \end{array}$$

*$\phi_{mn}\in \left[0,2\pi \right]$ is the angle the mth antenna on the reader makes with respect to the nth tag, where $\cos\phi_{mn}=\frac{{\bar{x}}_{m}-x_{n}}{d_{mn}}$ and $\sin\phi_{mn}=\frac{{\bar{y}}_{m}-y_{n}}{d_{mn}}$.*

**Proof.**  The Fisher Information Matrix (FIM) can be written as

(21) $$\begin{array}{c} J\left(p_{r}\right)=-E\left\{\frac{\partial U\left(p_{r}\right)}{\partial p_{r}}\right\}=\left[\begin{array}{cc} J_{11}\left(p_{r}\right) & J_{12}\left(p_{r}\right)\\ J_{21}\left(p_{r}\right) & J_{22}\left(p_{r}\right) \end{array}\right], \end{array}$$

where the gradient of the log-likelihood with respect to ${\bar{x}}_{m}$ and ${\bar{y}}_{m}$ is defined as

(22) $$U\left(p_{r}\right)=\left(\frac{\partial }{\partial {\bar{x}}_{m}}\log f_{p_{r}},\frac{\partial }{\partial {\bar{y}}_{m}}\log f_{p_{r}}\right).$$

The log-likelihood of the PDF of $f_{p_{r}}\left({\bar{P}}_{mn}^{f}\right)$ in (19) is described as

(23) $$\begin{array}{c} \log f_{p_{r}}\left({\bar{P}}_{mn}^{f}\right)=\sum_{m=1}^{M}\sum_{n=1}^{N}\left(\log\left(\frac{10}{\log10\sqrt{2\pi \sigma^{2}}{\bar{P}}_{mn}^{f}}\right)-\frac{b}{8}{\left(\log\frac{d_{mn}^{2}}{{\hat{d}}_{mn}^{2}}\right)}^{2}\right). \end{array}$$

Recall that $d_{mn}=\sqrt{{\left(x_{n}-{\bar{x}}_{m}\right)}^{2}+{\left(y_{n}-{\bar{y}}_{m}\right)}^{2}}$, the entries of (21) are obtained as

(24) $$J_{11}\left(p_{r}\right)=\sum_{m=1}^{M}\sum_{n=1}^{N}b{\left(\frac{{\bar{x}}_{m}-x_{n}}{d_{mn}^{2}}\right)}^{2}.$$

Using the relations $\cos\phi_{mn}=\frac{{\bar{x}}_{m}-x_{n}}{d_{mn}}$ and $\sin\phi_{mn}=\frac{{\bar{y}}_{m}-y_{n}}{d_{mn}}$, (24) is written as

(25) $$J_{11}\left(p_{r}\right)=\sum_{m=1}^{M}\sum_{n=1}^{N}b{\left(\frac{\cos\phi_{mn}}{d_{mn}}\right)}^{2}.$$

Following the same steps, the remaining elements are obtained as

(26) $$J_{22}\left(p_{r}\right)=\sum_{m=1}^{M}\sum_{n=1}^{N}b{\left(\frac{\sin\phi_{mn}}{d_{mn}}\right)}^{2},$$

and

(27) $$J_{12}\left(p_{r}\right)=J_{21}\left(p_{r}\right)=\sum_{m=1}^{M}\sum_{n=1}^{N}b\left(\frac{\cos\phi_{mn}\sin\phi_{mn}}{d_{mn}^{2}}\right).$$

The CRLB is just the inverse of FIM, and described as follows

(28) $$\mathrm{var}\left({\hat{p}}_{r}\right)\geq {\left\{J\left(p_{r}\right)\right\}}^{-1}.$$

Accordingly,

(29) $$\begin{array}{cc} \mathrm{var}\left({\hat{p}}_{r}\right) & \geq \frac{1}{|J\left(p_{r}\right)|}\left(J_{11}\left(p_{r}\right)+J_{22}\left(p_{r}\right)\right) \end{array}$$

(30) $$\begin{array}{c} \geq \frac{\sum_{m=1}^{M}\sum_{n=1}^{N}\frac{1}{d_{mn}^{2}}}{b\sum_{m=1}^{M}\sum_{n=1}^{N}\sum_{i=1}^{M}\sum_{k=1}^{N}Y_{n,m,i,k}}, \end{array}$$

where

$$\begin{array}{c} Y_{n,m,i,k}=\frac{\cos\phi_{mn}\sin\phi_{ik}\sin\left(\phi_{ik}-\phi_{mn}\right)}{d_{mn}^{2}d_{ik}^{2}}. \end{array}$$

 □

## 5. The Sub-Optimal Algorithms

Although the proposed algorithm results in the best WLLS estimation for the centroid of the antenna array, it has high computational complexity since lateration is performed for each antenna. Therefore, we propose in this part two sub-optimal algorithms aiming at reducing the computational complexity of the optimal algorithm. The proposed algorithms perform the lateration step only one time per location. Therefore, the computational complexity is reduced 1/M times compared to the optimal algorithm. The sub-optimal algorithms are proposed to estimate the position of the object benefiting from the available RSSI measurements according to the level of processing as follows: (1) distance-based averaging and (2) power-based averaging.

### 5.1. Distance-Based Averaging (Dis-Avg) Algorithm

The distance ${\tilde{d}}_{mn}$ can be approximated using small-angle approximations to be the distance between the reader and the *n*th tag ${\bar{d}}_{n}$, since the wavelengths of mm-wave radio signals are extremely short. After performing the ranging between the antennas on the reader and the *n*th tag, the distance vector ${\hat{d}}_{n}=\left({\tilde{d}}_{1n},{\tilde{d}}_{2n},..,{\tilde{d}}_{Mn}\right)$ is formed. Utilizing the *M* measurements contained in ${\hat{d}}_{n}$, the distance between the reader and the $n^{\mathrm{th}}$ tag can be approximated to be the average of the distance vector ${\hat{d}}_{n}$, which is expressed as

(31) $${\bar{d}}_{n}≃\frac{1}{M}\sum_{m=1}^{M}{\tilde{d}}_{mn}.$$

Consequently, the ranging between the reader and the DR tags can be accomplished forming the distance vector $\hat{d}=\left({\bar{d}}_{1},{\bar{d}}_{2},..,{\bar{d}}_{N}\right)$.

According to the derivation given in Appendix A, ${\bar{d}}_{n}$ can be approximated by a log-normal random variable with the parameters ${\bar{d}}_{n}$ ∼ $\mathrm{LN}({\hat{\mu }}_{n},{\hat{\sigma }}_{s})$, where

(32) $${\hat{\sigma }}_{s}=\log\left(\frac{\sum_{m=1}^{M}d_{mn}^{2}}{{\left(\sum_{m=1}^{M}d_{mn}\right)}^{2}}\left(e^{\sigma_{s}^{2}}-1\right)+1\right),$$

and

(33) $${\hat{\mu }}_{n}=\log\left(\frac{1}{M}\sum_{m=1}^{M}d_{mn}\right)+\frac{1}{2}\left(\sigma_{s}^{2}-{\hat{\sigma }}_{s}^{2}\right).$$

Afterwards, the lateration is executed for the reader. Thus, the lateration step is performed for $\hat{d}$ to estimate $p_{r}$. This can be modeled as

(34) $$H\;.\;p_{r}=\tilde{z},$$

where

(35) $$\tilde{z}=2\left[\begin{array}{c} x_{2}^{2}-x_{1}^{2}+y_{2}^{2}-y_{1}^{2}+{\bar{d}}_{1}^{2}-{\bar{d}}_{2}^{2}\\ \vdots \\ x_{N}^{2}-x_{1}^{2}+y_{N}^{2}-y_{1}^{2}+{\bar{d}}_{1}^{2}-{\bar{d}}_{N}^{2} \end{array}\right].$$

The model in (34) can be solved using WLLS estimator to find the estimate of $p_{r}$ as

(36) $${\hat{p}}_{r}={\left(H^{T}WH\right)}^{-1}H^{T}W\tilde{z},$$

where W is the weighting matrix calculated as the inverse of the covariance matrix of the vector $\tilde{z}$, which is described in (37), at the top of the next page.

(37) $$Q=\left[\begin{array}{cccc} \mathrm{var}\left({\bar{d}}_{1}^{2}\right)+\mathrm{var}\left({\bar{d}}_{2}^{2}\right) & \mathrm{var}\left({\bar{d}}_{1}^{2}\right) & \cdots & \mathrm{var}\left({\bar{d}}_{1}^{2}\right)\\ \mathrm{var}\left({\bar{d}}_{1}^{2}\right) & \mathrm{var}\left({\bar{d}}_{1}^{2}\right)+\mathrm{var}\left({\bar{d}}_{3}^{2}\right) & \cdots & \mathrm{var}\left({\bar{d}}_{1}^{2}\right)\\ \vdots & \vdots & \ddots & \vdots \\ \mathrm{var}\left({\bar{d}}_{1}^{2}\right) & \mathrm{var}\left({\bar{d}}_{1}^{2}\right) & \cdots & \mathrm{var}\left({\bar{d}}_{1}^{2}\right)+\mathrm{var}\left({\bar{d}}_{N}^{2}\right) \end{array}\right]$$

Therefore, according to (8), the variance of ${\bar{d}}_{n}^{2}$ is written as

(38) $$\begin{array}{cc} \mathrm{var}\left({\bar{d}}_{n}^{2}\right) & =e^{{\hat{\mu }}_{n}^{4}}\left(e^{8{\hat{\sigma }}_{s}^{2}}-e^{4{\hat{\sigma }}_{s}^{2}}\right). \end{array}$$

As discussed before, the lateration is performed only one time for the approximated averaged distance, which reduces the computational complexity of localization.

### 5.2. Power-Based Averaging (Power-Avg) Algorithm

In this sub-optimal algorithm, the received backscattered power at the centroid of the reader corresponding to the *n*th tag is approximated to be the average of the received signals at the different antennas and expressed as

(39) $${\bar{P}}_{n}^{f_{n}}=\frac{1}{M}\sum_{m=1}^{M}{\bar{P}}_{mn}^{f_{n}}.$$

This approximation is based on the fact that all the antennas are part of the reader, and the received power at any antenna is related to the position of the reader itself. Therefore, the multiple RSSI measurements available at the reader are averaged out to overcome the effect of the channel fading. Accordingly, the distance between the centroid of the reader and the *n*th tag, i.e., ${\bar{d}}_{n}$, can be estimated from ${\bar{P}}_{n}^{f_{n}}$ according to the considered channel model to be ${\bar{d}}_{n}={\left(\frac{{\bar{P}}_{n}^{f_{n}}}{{\bar{P}}_{d_{o}n}^{f_{n}}}\right)}^{\frac{-1}{2\alpha }}$. As a consequence, the distance vector $\bar{d}=\left({\bar{d}}_{1},{\bar{d}}_{2},..,{\bar{d}}_{N}\right)$ can be obtained in the same manner. From the channel model in (4), the measured power ${\bar{P}}_{mn}^{f_{n}}$ follows a log-normal random distribution with the parameters ${\bar{P}}_{mn}^{f_{n}}∼\mathrm{LN}\left({\bar{\mu }}_{mn},{\bar{\sigma }}_{s}\right)$, where

(40) $${\bar{\mu }}_{mn}=\log\left(\frac{{\bar{P}}_{d_{o}n}^{f_{n}}}{{\left(d_{mn}\right)}^{2\alpha }}\right),$$

and

(41) $${\bar{\sigma }}_{s}=\frac{\sigma \log10}{10}.$$

According to the derivation given in Appendix A, ${\bar{P}}_{n}^{f_{n}}$ can be approximated by a log-normal random variable with the parameters ${\bar{P}}_{n}^{f_{n}}$ ∼ $\mathrm{LN}({\overset{˘}{\mu }}_{n},{\overset{˘}{\sigma }}_{s})$, where

(42) $${\overset{˘}{\sigma }}_{s}=\log\left(\frac{\sum_{m=1}^{M}{\left(d_{mn}\right)}^{-4\alpha }}{{\left(\sum_{m=1}^{M}{\left(d_{mn}\right)}^{-2\alpha }\right)}^{2}}\left(e^{{\bar{\sigma }}_{s}^{2}}-1\right)+1\right),$$

and

(43) $${\overset{˘}{\mu }}_{n}=\log\left(\frac{1}{M}\sum_{m=1}^{M}\frac{{\bar{P}}_{d_{o}n}^{f_{n}}}{{\left(d_{mn}\right)}^{2\alpha }}\right)+\frac{1}{2}\left({\bar{\sigma }}_{s}^{2}-{\overset{˘}{\sigma }}_{s}^{2}\right).$$

The last step is to execute the lateration algorithm for the distance vector $\bar{d}$ to estimate $p_{r}$ by following the steps in (34) to (36). The weighting matrix W for WLLS estimator can be formed as in (37), where it mainly depends on the variance of ${\bar{d}}_{n}^{2}$ as follows

(44) $$\begin{array}{cc} \mathrm{var}\left({\bar{d}}_{n}^{2}\right) & =E\left[{\bar{d}}_{n}^{4}\right]-{\left(E\left[{\bar{d}}_{n}^{2}\right]\right)}^{2}\\ \; & =E\left[{\left(\frac{{\bar{P}}_{n}^{f_{n}}}{{\bar{P}}_{d_{o}n}^{f_{n}}}\right)}^{\frac{-2}{\alpha }}\right]-{\left(E\left[{\left(\frac{{\bar{P}}_{n}^{f_{n}}}{{\bar{P}}_{d_{o}n}^{f_{n}}}\right)}^{\frac{-1}{\alpha }}\right]\right)}^{2}\\ \; & ={\left({\bar{P}}_{d_{o}n}^{f_{n}}\right)}^{\frac{\alpha }{2}}\left(E\left[{\left({\bar{P}}_{n}^{f_{n}}\right)}^{\frac{-2}{\alpha }}\right]-{\left(E\left[{\left({\bar{P}}_{n}^{f_{n}}\right)}^{\frac{-1}{\alpha }}\right]\right)}^{2}\right)\\ \; & ={\left({\bar{P}}_{d_{o}n}^{f_{n}}\right)}^{\frac{\alpha }{2}}e^{\frac{2{\overset{˘}{\sigma }}_{s}^{2}}{\alpha }\left(\frac{1}{\alpha }-{\overset{˘}{\mu }}_{n}\right)}\left(e^{{\overset{˘}{\sigma }}_{s}^{2}}-1\right). \end{array}$$

## 6. Measurements and Simulation Setup

This section considers the measurement validation of the DR tag and a realistic modeling of the path loss for an indoor environment using 3D ray-tracing. The system is operated in the 63 GHz frequency range. Unless otherwise stated, the reader has a planer array of 10×5 antennas (M=50), where the gain of the single element at the reader is 2.15 dBi. In the considered indoor area, many reference DR tags are uniformly located on the ceiling of the room in order to achieve the full coverage in the considered area and to enable the reader localizing itself using WLLS RSSI algorithm. However, the reader is assumed to be connected only for N=8 reference tags to perform localization. All measurements and simulation parameters are included in Table 1.

### 6.1. Measurements

**Figure 4:**
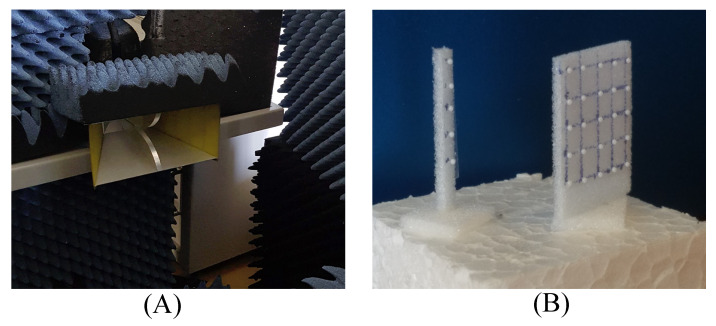
The measurement setup. (**A**) Double ridge horn. (**B**) 5×5 and 5×1 DR array configurations.

In this section, the experimental verification is presented for the large-scale-based DR tag based on the monostatic RCS of 5×5 and 5×1 element planar DR arrays with element spacing of λ/2 at 63 GHz (0.8 mm diameter, manufactured by T-Ceram, Czech Republic). The scattering parameter S11 was measured employing an HP8510 Vector Network Analyzer (VNA) with the HP85104A mm-wave converter for the 50–75 GHz range, equipped with a 25 dBi standard gain horn antenna. The DR array was placed on a block of Styrofoam ($\epsilon_{r}=1.04$) in about 60 cm axial distance from the horn aperture. Due to the very low level of the scattered power from the DR array, we used background subtraction to make it visible. First, the scattering coefficient without the DR array in place was recorded and afterwards subtracted from the scattering coefficient, which was measured with the DR in place. The conversion of the measured reflection coefficient data of the DR array to RCS magnitudes was performed by normalization to the reflection coefficient of a metal sphere of 20 mm diameter metal at the center of the band and multiplication with its RCS magnitude; since the frequency-dependent gain of the horn antenna is not corrected for, as in the method presented in [74], the RCS magnitudes provided by this method are only approximate. Figure 4 shows the measurement setup.

**Figure 5:**
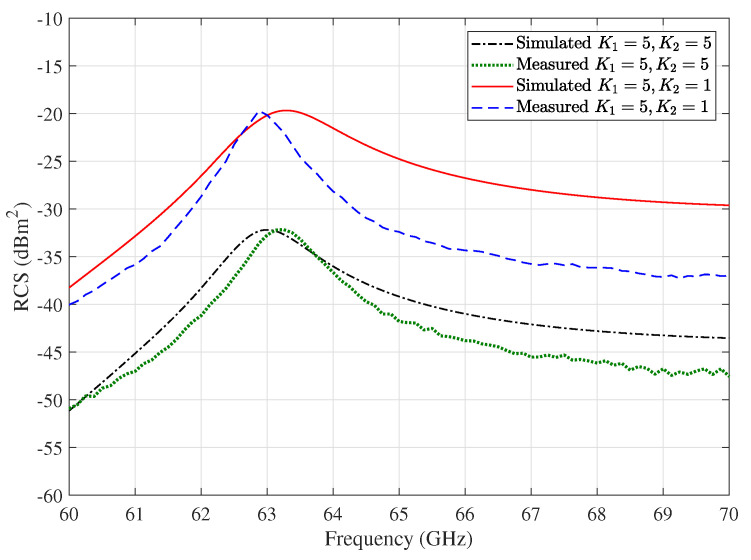
Measured RCS magnitudes compared to simulated RCS for 5×5 and 5×1 DR array structures.

Figure 5 shows a comparison of the measured RCS to the simulation results at the first resonant frequency. Agreement between measured and simulated monostatic RCS is found across the full frequency range.

### 6.2. Localization Coverage Area

We investigated the impact of the severe path loss on the ranging coverage area. Accordingly, this coverage area is characterized using the path loss threshold $L_{\mathrm{th}}$, which is defined as [2]

(45) $$\begin{array}{c} 2L_{\mathrm{th}}\left(d_{mn},f\right)\left[dB\right]=P_{T}\left[dB\right]+G_{R}\left[dB\right]+{\bar{G}}_{R}\left[dB\right]+G_{P}\left[dB\right]+10\log\left(\frac{4\pi \rho_{n}^{2}\sigma_{n}}{\lambda^{2}}\right)-P_{w}-\gamma , \end{array}$$

where $G_{P}$ is the signal processing gain, and γ is the noise figure and other channel and hardware impairments. The noise power is described as

(46) $$\begin{array}{c} P_{w}=10\log_{10}\left(kTB\right)\;\left[\mathrm{dBW}\right], \end{array}$$

where *k* is the Boltzmann constant, *T* is the temperature in Kelvin, and *B* is the bandwidth. The coverage area is specified to have path loss smaller than the threshold.

**Figure 6:**
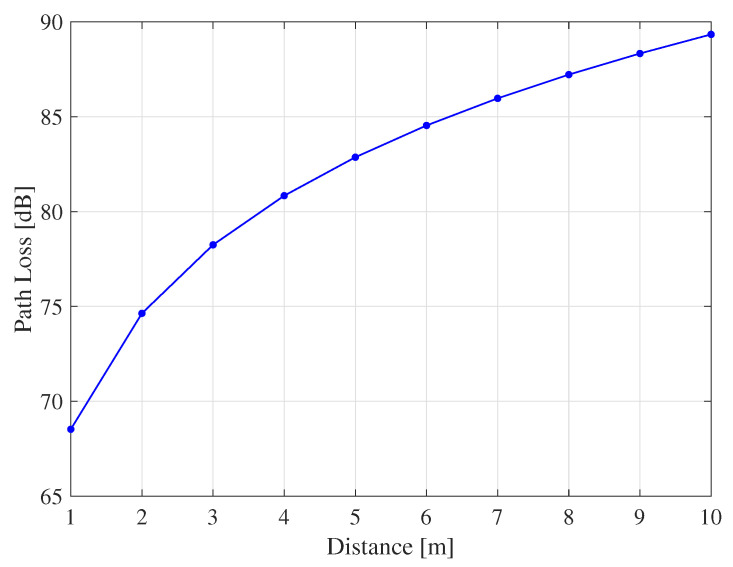
Coverage area of RFID localization system at operating frequency f=63 GHz.

Assuming the resonance frequency is f=63 GHz, $G_{P}=10$ dB, and γ=10 dB, the path loss threshold is calculated to be around 83 and 77 dB for 5×5 and 5×1 DR tags structures, respectively. This means that the reader can detect a DR tag as far as up to 5.2 m and 2.6 m as shown from Figure 6 for the 5×5 and 5×1 DR tags structures, respectively. Next, the 5×5 DR tag is considered due to its better range coverage.

### 6.3. Path Loss Model Using 3D Ray-Tracing

Since real-world mm-wave measurements are time intensive and expensive, Wireless InSite (WI) is used as a 3D ray-tracing [75]. It was verified that modeling the propagation channel can be provided using 3D ray-tracing with high accuracy [76,77,78]. Moreover, an excellent agreement with measurements for narrowband and wideband wireless channels was shown in the literature. This model considers the spatial channel and the environmental effects as path-loss, frequency dependence, scattering, reflections, transmissions, and diffractions. It considers as well the characteristics of the antennas as part of the effective channel such as directional gain, matching, and polarization.

**Figure 7:**
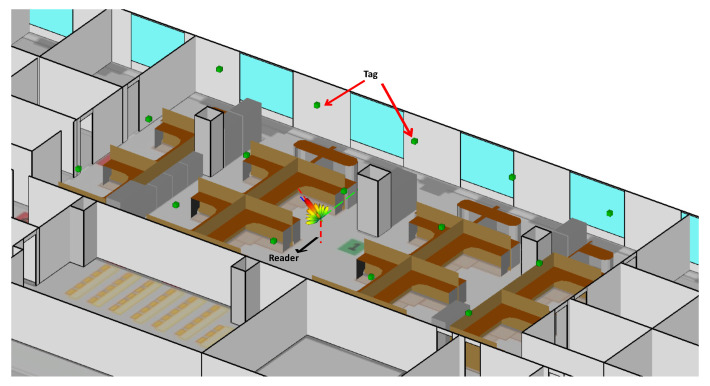
3D layout of simulated real environment of the office room modeled in Wireless InSite (WI).

The office environment of a 25 × 12 × 3 m area is modeled as seen in Figure 7. The precise geometry of the office environment was entered into the Ray-Tracer following the ITU recommendations as described in one of the examples in [75]. The reference tags are nested at the ceiling of the indoor environment as shown Figure 7. As described in Section 3.3, beamforming is executed in the transmitting mode contrary to the receiving mode, where there is no favored receiving direction at receiving mode, and the signals across the array elements are not combined. Moreover, and in order to consider the properties of the designed tag in channel realizations, the tag designed in Section 6.1 using CST is exported to WI in addition to the reader antenna array.

The deterministic channel realizations are collected using 3D ray tracing for random positions by moving the reader in this environment randomly.

## 7. Results and Discussion

Here, we evaluate the proposed large-scale MIMO-based localization algorithm using the deterministic and analytical channels. The optimal algorithm is denoted next by the *L-MIMO-RSSI* algorithm. Moreover, the sub-optimal algorithms are evaluated compared to the *L-MIMO-RSSI* algorithm in order to examine their localization accuracies. The performances of the proposed algorithms are compared with a reference system denoted by *Ref* algorithm, in which the reader is equipped with only one antenna. All the simulation parameters are included in Table 1.

The reader sweeps the frequency band 5–63 GHz in order to find the reference tags within its coverage. Such process could be executed using a pulse centered at 60 GHz of bandwith 6 GHz or using a frequency modulation signal sweeping the frequency band during the t-second pulse duration. After that, the detected tags with the maximum peak resonance are chosen, where the reader sequentially transmits signals that are centered at their resonance frequencies and cover the resonance bandwidth. The RSSIs of the backscattered signals from these tags are post-processed to perform ranging and, then, lateration.

For the sake of comparison, the cumulative distribution function (CDF) curves of the root-mean-square error (RMSE) of localization error are considered, where RMSE is written as

(47) $$e_{\mathrm{rmse}}=\sqrt{E\left\{{\left(\hat{x}-\bar{x}\right)}^{2}+{\left(\hat{y}-\bar{y}\right)}^{2}\right\}}.$$

### 7.1. Analytical Channels

In order to evaluate the proposed algorithm for different environments and scenarios, the analytical channels have been generated using the channel model described in (4) within a room of dimensions $w_{1}=w_{2}=10$ m. The work in [64] intensively studies the path loss models in mm-wave band and concludes that the CI log-normal path is efficient as it offers the simple and accurate calculation for path loss. Moreover, this work presents the modeling of CI model parameters using measurements. Therefore, we utilized the results of [64] in our work and reproduced it for 63 GHz. Although the passive RFID system is more reliable and robust in LOS environments, we considered in our evaluation of the proposed algorithm NLOS indoor environments in addition to LOS environments for co-polarization, cross-polarization, and combined-polarization antenna configurations. In order to delete the outlier measurements, a modified Thompson Tau test was utilized to detect outliers as proposed in [27].

This part evaluates the proposed algorithm in the LOS environment using different antenna configurations. For this scenario, α and σ are predicted from [64] to be respectively around 1.62 and 3 dB for the co-polarized antenna configuration, 4.5 and 8.4 dB for the cross-polarized case, and 3 and 16 dB for the combined-polarization case.

**Figure 8:**
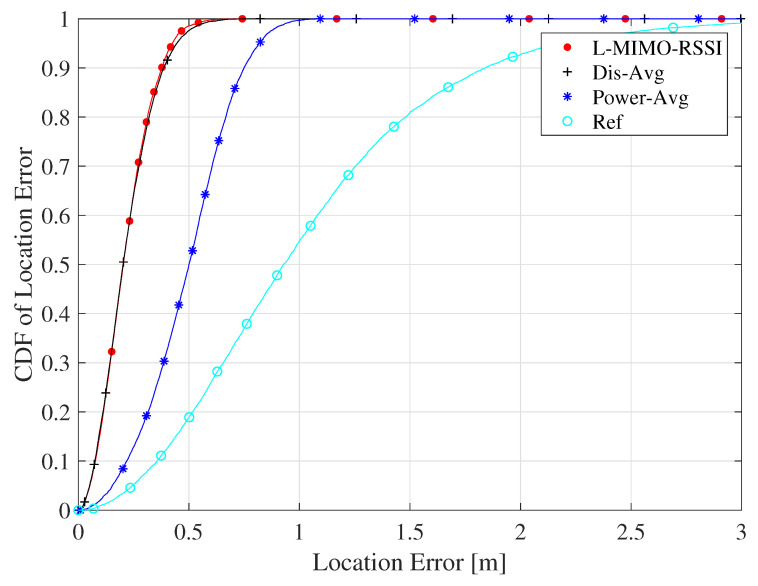
The cumulative distribution function (CDF) of localization root-mean-square error (RMSE) for the proposed algorithms for the line-of-sight (LOS) environment and the co-polarized antenna configuration.

Figure 8 depicts the CDF of the location error for the proposed algorithms compared to the reference system for the case of co-polarized antenna configuration. The *L-MIMO-RSSI* algorithm achieved the best localization performance yielding a maximum location error of 36 cm at 90% confidence, while the reference system had 1.9 m at 90% confidence. This illustrates the significant localization accuracy improvement that can be achieved using the proposed algorithm. The *Dis-Avg* algorithm closely approached the *L-MIMO-RSSI* algorithm with 1/M lower computational complexity. On the other side, the *Power-Avg* sup-optimal algorithm had a maximum of 75 cm location error at 90% confidence. This figure clearly shows that the *Dis-Avg* algorithm achieved the required performance similar to the *L-MIMO-RSSI* algorithm with lower complexity.

**Figure 9:**
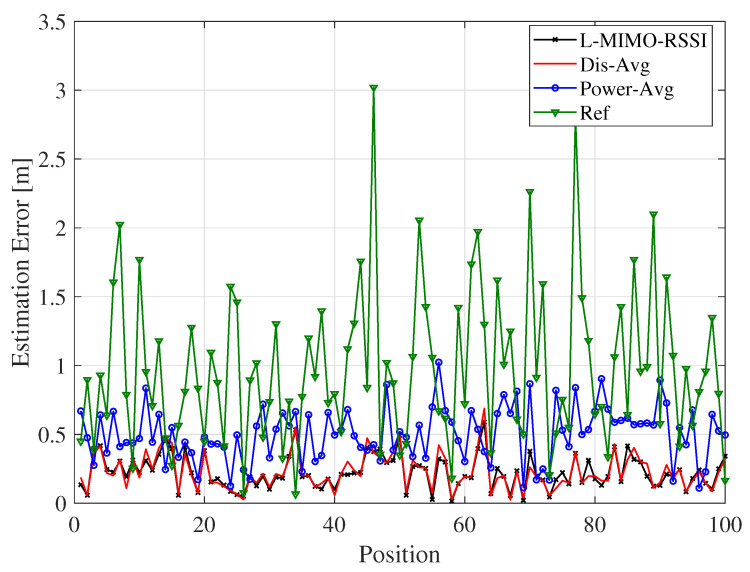
Localization errors change with respect to different random object positions for the LOS environment and the co-polarized antenna configuration.

Figure 9 plots the instantaneous estimation error with respect to random object positions for the proposed algorithms for the same case of Figure 8. It is noted from the figure that the instantaneous estimation error fluctuated with the position changes due to the multipath propagation. The fluctuations of the reference algorithm were much stronger, which caused deep estimation error degradation at some positions. However, *L-MIMO-RSSI*, *Dis-Avg*, and *Power-Avg* algorithms presented smooth instantaneous estimation errors compared to the reference system, which means that the large-scale MIMO-based algorithms can decrease the effect of small-scale fast fading and improve the localization accuracy. Figure 8 also confirms that *L-MIMO-RSSI* and *Dis-Avg* algorithms behaved similarly and achieved the best performance.

**Figure 10:**
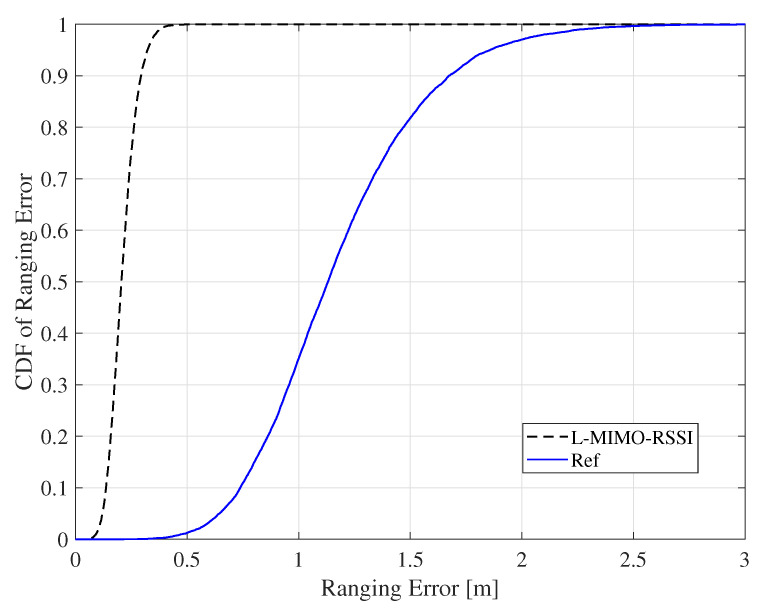
The CDF of ranging RMSE for the proposed algorithm for the LOS environment and the co-polarized antenna configuration.

Figure 10 depicts the CDF of the ranging error for the proposed algorithm compared to the reference system for the case of co-polarized antenna configuration. The *L-MIMO-RSSI* algorithm achieved much better ranging accuracy yielding a maximum location error of 29 cm at 90% confidence, while the reference system had 1.65 m at 90% confidence. This illustrates the significant ranging accuracy improvement that can be achieved using the proposed algorithm, which reflects on the localization accuracy improvement.

**Figure 11:**
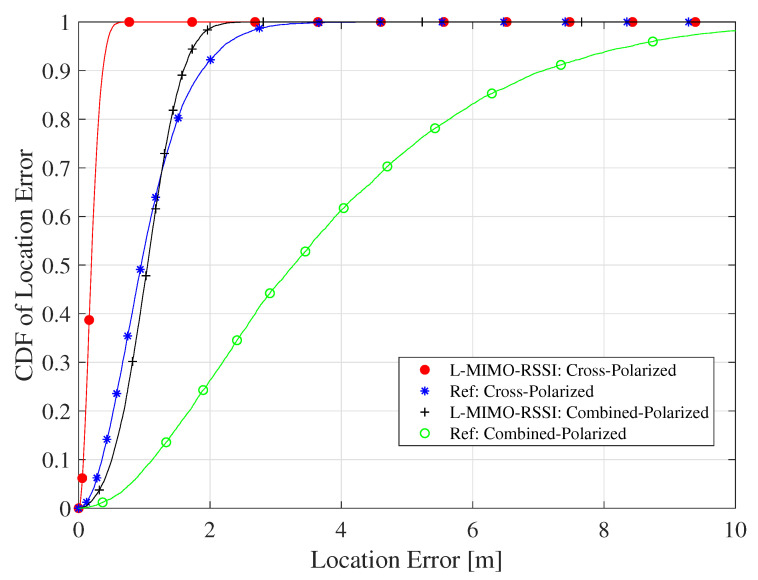
The CDF of localization RMSE for the proposed algorithm for the LOS environment and the cross- and combined polarized antenna configurations.

Figure 11 shows the CDF of the location error for the proposed algorithm for the cases of the cross-polarized and combined-polarized antenna configurations. The *L-MIMO-RSSI* algorithm highly improved the localization performance achieving a maximum location error of 37 cm and 1.5 m at 90% confidence, while the reference system had 90 cm and 7 m at 90% confidence for the cross- and combined-polarized cases, respectively. It is worth mentioning here that even the localization accuracy at the combined-polarized case was worse compared to the other cases; the amount of improvement compared to the reference system was much larger than that of the other cases. In other words, RSSI localization in the combined-polarized case does not work without our proposed algorithm, which guides RSSI localization to achieve acceptable localization accuracy.

**Figure 12:**
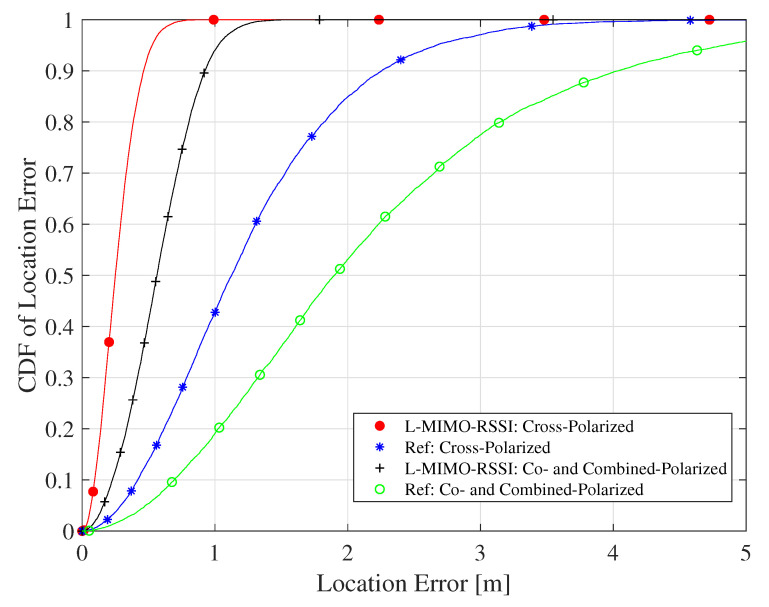
The CDF of localization RMSE for different position estimation algorithms for the NLOS environment and the co-, cross- and combined-polarized antenna configurations.

In order to evaluate the proposed algorithm in the NLOS environment, α and σ are predicted from [64] to be, respectively, around 3.3 and 11.5 dB for the co- and combined-polarized antenna configurations and 4.8 and 10.5 dB for the cross-polarized case. Figure 12 shows the CDF of the location error performance for the proposed algorithm for these cases. Here, the proposed algorithm also presents efficient performance, where maximum location errors of 64 cm for the co- and combined cases and 91 cm for the cross-polarized case were achieved at 90% confidence, while the reference system had 2.2 and 4 m, respectively.

We further investigated the system and environmental parameters on the proposed algorithm using the analytical channels for the LOS environment using the co-polarized antenna configuration.

**Figure 13:**
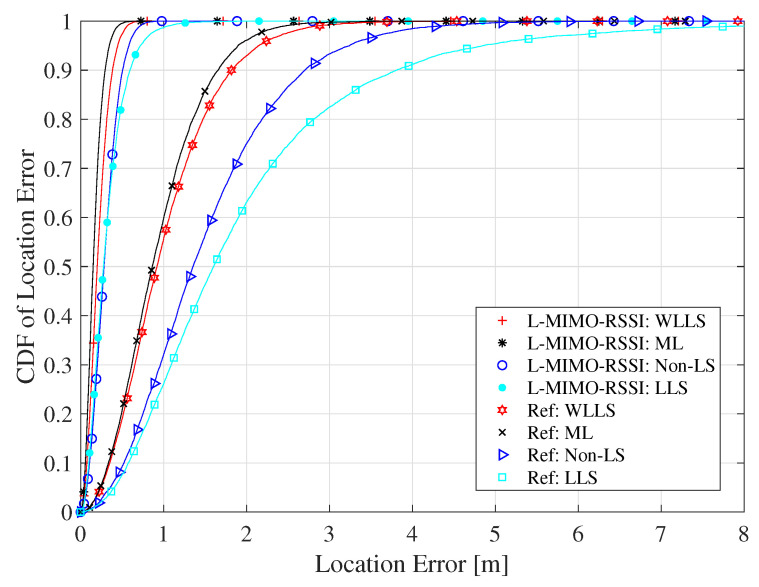
The CDF of localization RMSE for different position estimation algorithms for the LOS environment and the co-polarized antenna configuration.

We studied the performance of the localization algorithms using different position estimators, where WLLS, ML [70], Non-LSE [72], and LLS are considered. WLLS is simulated as discussed in Section 4, while the ML approach is implemented using the Levenberg–Marquardt method [70] with a damping factor of 10 and 20 iterations. The resulting CDF of the location error is presented in Figure 13. We notice that large-scale MIMO-based localization framework was highly satisfactory in improving the localization accuracy compared to the reference algorithm using the considered estimators. Even though ML achieved the more robust computation of location estimates compared to WLLS, LLS, and Non-LS schemes, the ML method involves extensive calculations making its implementation unfeasible for real time localization. WLLS performed very close to ML with much lower computational complexity. It is worth mentioning here that the LLS algorithm gains considerably more benefit from large-scale MIMO compared to the other estimators, where the CDF of the location error difference between large-scale MIMO-based LLS and WLLS algorithm is 26 cm at 90% confidence, whereas this difference for the single antenna system is 2 m at the same confidence level.

**Figure 14:**
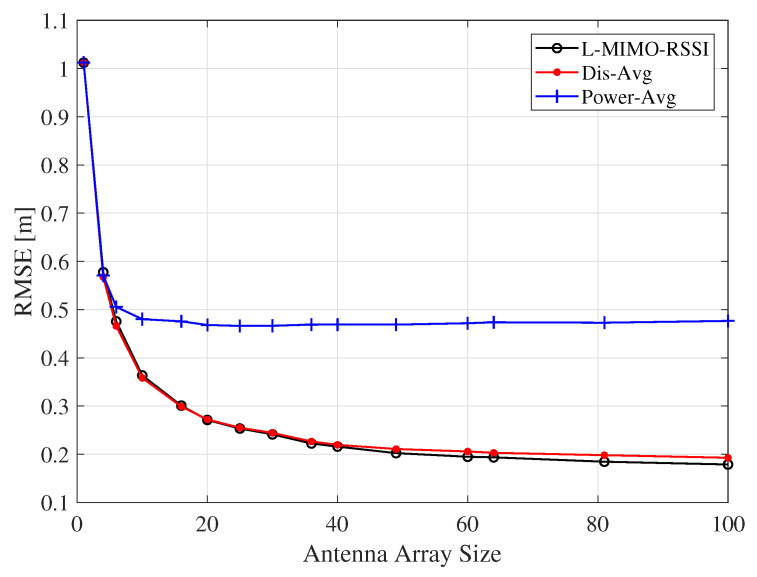
The localization RMSE for the proposed algorithms for different number of antennas on the reader for the LOS environment and the co-polarized antenna configuration.

The influences of antenna array size of the reader on the RMSE of localization accuracy is shown in Figure 14. We can observe from this figure that the RMSE of the localization error decreased by adding more antennas at the reader side. It is noted that the significant improvement in localization accuracy appears when the number of antennas is enlarged from 1 to 30, where RMSE degrades from 1 m to 24 cm for the *L-MIMO-RSSI* algorithm. Moreover, this figure shows that the *Dis-Avg* algorithm behaved very similarly to the *L-MIMO-RSSI* algorithm and outperformed the *Power-Avg* algorithm.

**Figure 15:**
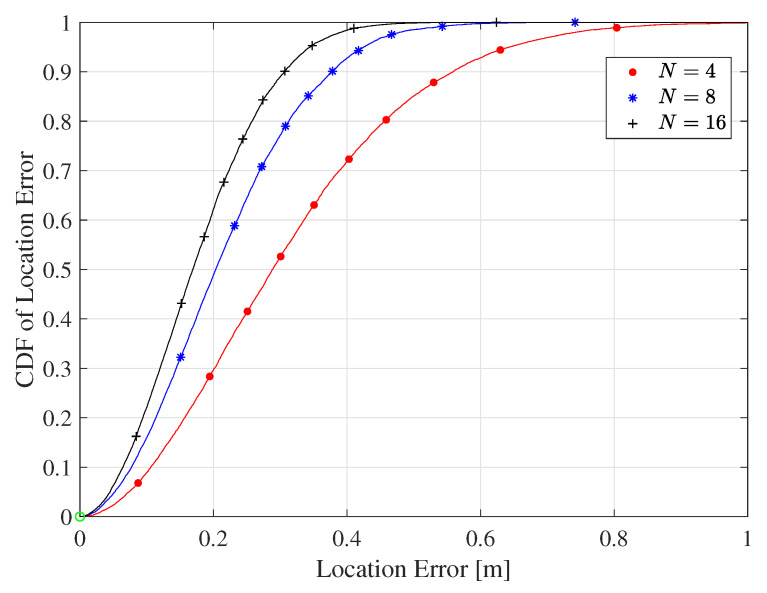
The CDF of the RMSE for different numbers of nested reference tags for the LOS environment and the co-polarized antenna configuration.

Figure 15 evaluates the impact of the number of connected DR tags to the reader on the localization accuracy, which shows that the localization accuracy improved as the number of reference tags increased, reaching 30 cm at 90% confidence when N=16.

**Figure 16:**
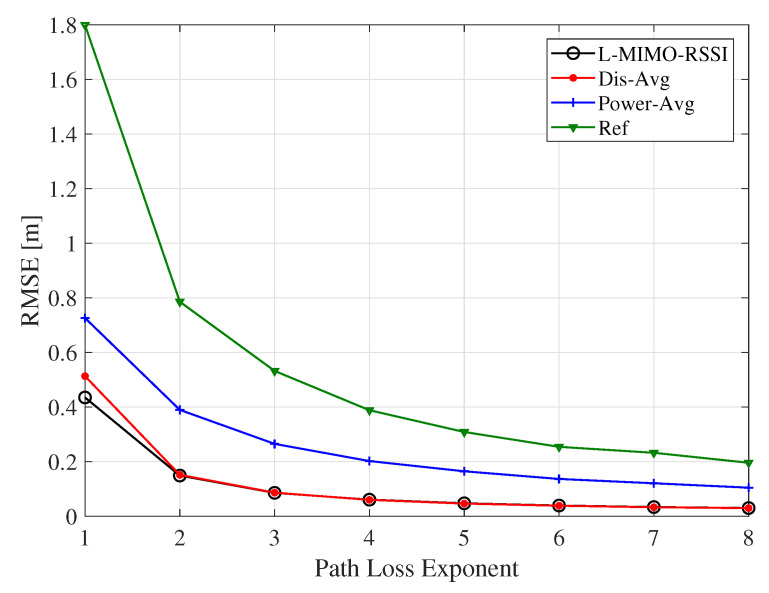
The RMSE for the proposed algorithms for different path loss exponent values. *M* = 50, *N* = 8, σ = 3.

The effect of the surrounding environment is characterized by the factor α in the log-normal path loss model, which can vary over a large range [57,63,64]. Therefore, the effect of the path loss exponent on the localization accuracy is explored by simulating the RMSE of the proposed algorithms for different values of α for the system configuration M=50, N=8 and σ = 3. The simulation result is illustrated in Figure 16, where we observe that the localization RMSE decreased with the growth of the path loss exponent. Moreover, this figure exhibits that large-scale MIMO-based localization algorithms were more robust against the change of α compared to the reference system. The RMSE of the reference system changed from 1.8 to 0.2 m when α changed from α=1 to α=8, while RMSE of the *L-MIMO-RSSI* algorithm changed within the range of 43 to 3 cm for the same path loss exponent change range.

**Figure 17:**
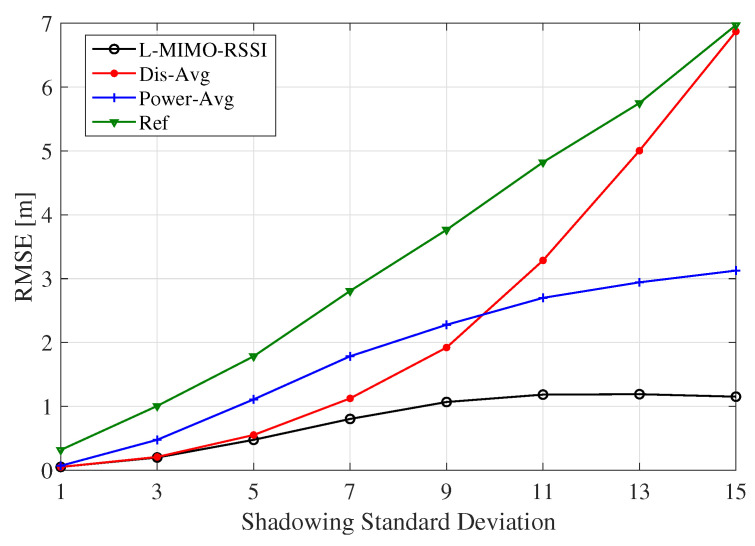
The RMSE for the proposed algorithms for different standard deviation values of shadowing. *M* = 50, *N* = 8, α = 1.62.

Figure 17 exhibits the effect of the shadowing on localization accuracy when M=50, N=8, and α=1.62. As expected, as the standard deviation of the shadowing increased, the RMSE of the localization accuracy worsened. However, the *L-MIMO-RSSI* algorithm presented more immunity against the shadowing. However, the simplified algorithm *Dis-Avg* was highly degraded for larger values of σ due to the averaging before performing localization, which leads to high error in the lateration step.

**Figure 18:**
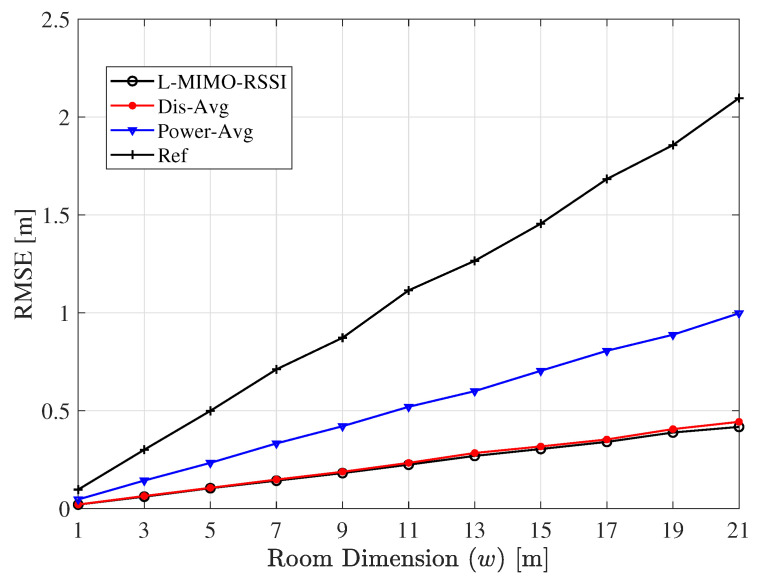
The RMSE for the proposed algorithms for different indoor area dimensions $w_{1}=w_{2}=w$. *M* = 50, *N* = 8, σ = 3, α = 1.62.

The influence of the network size on the localization accuracy is investigated in Figure 18 for different room dimensions $w_{1}=w_{2}=w$. The simulation is performed for LOS and co-polarized scenarios with M=50 and N=8. Although the estimation error degraded with the enlargement of the room size, the *L-MIMO-RSSI* algorithm presented more privilege against the increase of the network size.

**Figure 19:**
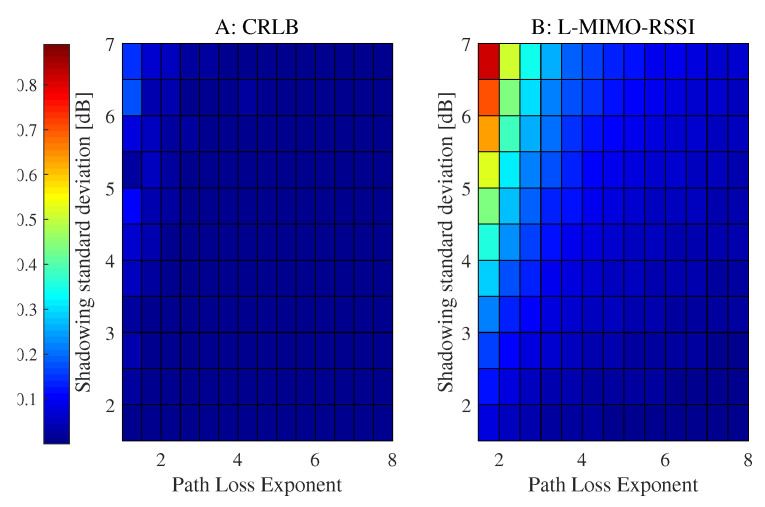
Cramér–Rao Lower Bound (CRLB) for different path loss exponent and shadowing standard deviation values.

Figure 19 presents the comparison between the proposed algorithm and the CRLB for different path loss exponent and shadowing standard deviation values. It is noted that the proposed algorithm approached the CRLB in most cases, except when the path loss exponent was below 2 and the the shadowing standard deviation was greater than 5; to the best of our knowledge, this case was not observed in the literature. The measurements in the literature [64] conclude that at LoS scenarios, i.e., path loss exponent below 2, the shadowing standard deviation is also below 5 dB. Therefore, we conclude that the proposed algorithm is efficient since it presents also a close performance to CRLB.

**Figure 20:**
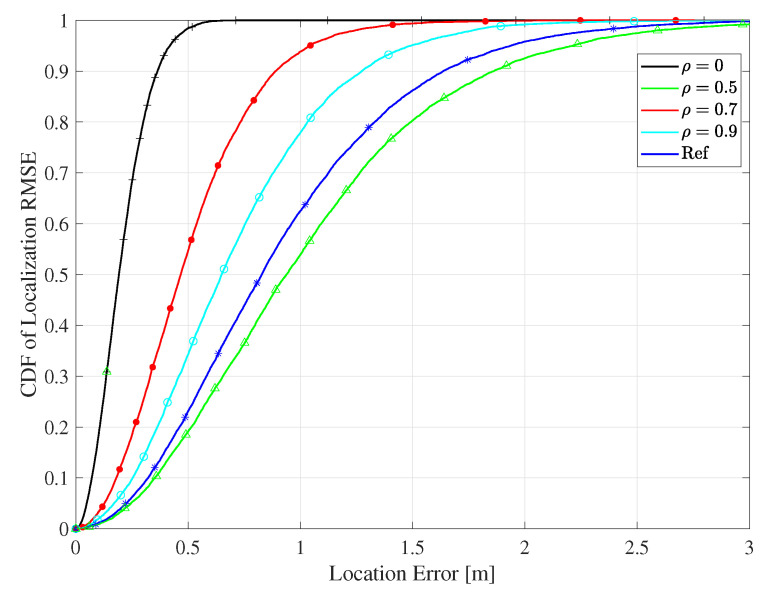
The CDF of the RMSE for the proposed algorithms for different spatial correlation values ρ for the LOS environment and the co-polarized antenna configuration.

Figure 20 shows the effect of spatial correlation between the reader antennas on the RMSE performance for the proposed algorithms for the LOS environment and the co-polarized antenna configuration, when the reader has *M* = 50 antennas, and 8 reference tags are distributed in the environment. Each antenna element on the reader was spatially correlated to the adjacent one by the factor ρ. The existence of spatial correlation affected adversely the orthogonality of the channels and, hence, the channel favorability and hardening of large-scale MIMO. As a consequence, the shadowing was not well averaged as the case of the orthogonal channels leading to the degradation in localization accuracy. Although a spatial correlation existed, the proposed algorithm improved the localization accuracy compared to the conventional system.

Compared with the recent and related works in [16,17,31], the proposed algorithm achieved significant localization accuracy improvement, where RMSE can reach 16 cm in a room with dimensions 8×8 m when M=50 and N=8. The work in [16] achieved 1 m at 82% confidence in a room with dimensions 8×8 m using the best proposed configuration, that is a bistatic reader with a π/2 antenna elevation angle and with the antennas placed towards the center of the side walls, when the transmitted power equals 3 W. Similarly, the work in [17] achieved precision of about 90% within 0.5 m in a room with dimensions 6.5×10 m using grid-based pattern of many reference tags. The work in [31], which considers RSSI at mm-wave band, showed that it is possible to achieve an accuracy of around 1 m in a room with dimensions 8.9×16 m if a sufficient number of measurement samples is acquired. However, and for approximately the same area of the work in [31], our proposed approach achieved an accuracy of 35 cm. Furthermore, the work in [79] summarizes many RSSI algorithms, and our proposed algorithm outperformed the mentioned algorithms in this work.

### 7.2. Deterministic Channels

We present here the performance of the proposed algorithm using deterministic channels. These channels are collected as described in Section 6.3.

**Figure 21:**
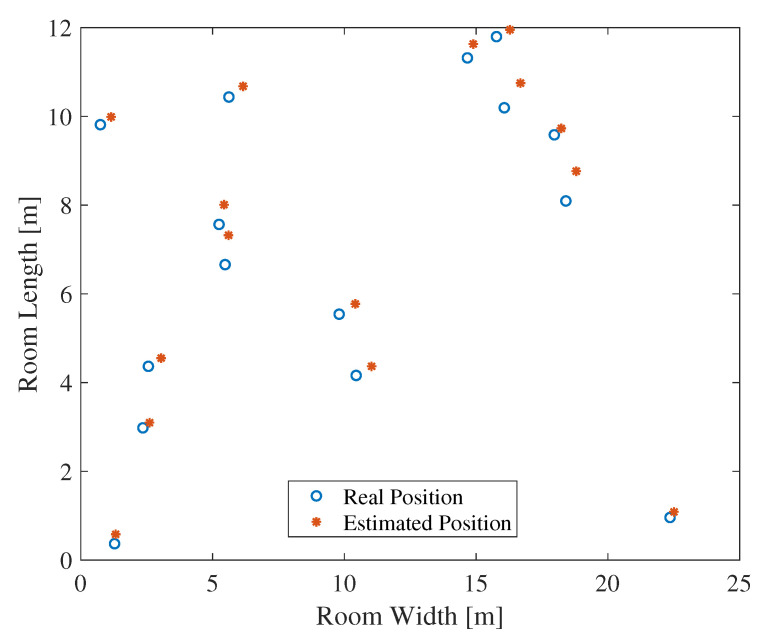
Estimated positions of the reader using deterministic channels, where the *L-MIMO-RSSI* algorithm is used.

In order to evaluate the proposed algorithm at different locations in the room, the reader was allocated at 15 random positions while the tags were placed at fixed positions, as shown in Figure 7. The reader was connected to the nearest five tags in order to perform localization using the proposed algorithm. Figure 21 depicts the estimated and real positions of the reader in the considered area. The average RMSE of the localization estimation was around 75 cm, where a minimum of 25 cm was observed.

## 8. Conclusions

In this paper, we proposed RSSI-based localization with low-complexity DR RFID infrastructure utilizing the potential of large-scale MIMO technology operated at mm-wave band in order to enhance the self-localization accuracy. DR tags are designed to act as reference anchors for the infrastructure, where each DR tag is composed from an array of DR elements with unique resonance frequencies in order to improve the detectability of the tag and reduce the inter-tag interference. The object to be localized is also equipped with large-scale antennas in order to reduce the sensitivity of the localization accuracy against channel fading, which improves the capability of smart objects in estimating their positions. RSSI as a simple ranging algorithm, and WLLS lateration algorithms are considered in the proposed algorithm. The proposed algorithm first estimates the position of each antenna on the object and then finds the centroid point of the antenna array to be the position of the object. The proposed framework can be combined with most position estimation techniques. In this work, we have applied the WLLS estimation technique to the proposed large-scale MIMO framework. The CRLB on the localization accuracy is derived taking into account the large-scale MIMO on the object. To decrease the computational complexity of performing lateration for all the antennas, two sub-optimal algorithms are proposed to estimate the position of the object.

Measurement results are presented to validate the design of the reference DR tags concept. Moreover, simulation results showed that the proposed algorithm improved the accuracy of localization. The *Dis-Avg* algorithm was able to achieve a performance very close to that of the optimal algorithm with much lower computational complexity, so it is recommended to be used to substitute the optimal algorithm. It is noted that the proposed large-scale MIMO framework is applicable to different position estimation techniques. Furthermore, simulation results showed that the proposed algorithms were robust against the channel fading and environment changes.

Succeeding research will realize and evaluate this work in a real environment using the designed infrastructure and IoT devices.

## Figures and Tables

**Table 1:** Large-scale MIMO-based RFID localization system parameters.

Parameter	Value
Frequency Range	57–63 GHz
Operating Bandwidth	100 MHz
Transmit Power $P_{T}$	10 dBm
Reader Antenna Element Gain $G_{T}$	2.15 dBi
Room Width and Length	10×10 $m^{2}$

## Appendix A

In this Appendix, we derive the distribution of the sum of general *K* independent log-normal random variables by using the Fenton–Wilkinson approximation [80]. Assume a variable $y_{k}$ follows a log-normal distribution as $y_{k}∼\mathrm{LN}\left({\tilde{\mu }}_{k},\tilde{\sigma }\right)$. Define

(A1) $$y=\sum_{k=1}^{K}{\tilde{y}}_{k}.$$

According to Fenton–Wilkinson approximation, *y* can be approximated by a log-normal random variable, i.e., *y* ∼ $\mathrm{LN}(\overset{`}{\mu },\overset{`}{\sigma })$, where $\overset{`}{\mu }$ and $\overset{`}{\sigma }$ satisfy

(A2) $$\sum_{k=1}^{K}e^{\left({\tilde{\mu }}_{k}+\frac{{\tilde{\sigma }}^{2}}{2}\right)}=e^{\left(\overset{`}{\mu }+\frac{{\overset{`}{\sigma }}^{2}}{2}\right)},$$

and

(A3) $$\sum_{k=1}^{K}e^{2{\tilde{\mu }}_{k}}\left(e^{2{\tilde{\sigma }}^{2}}-e^{{\tilde{\sigma }}^{2}}\right)=e^{2\overset{`}{\mu }}\left(e^{2{\overset{`}{\sigma }}^{2}}-e^{{\overset{`}{\sigma }}^{2}}\right).$$

By solving (A2) for $\overset{`}{\mu }$, (A2) can be written as

(A4) $$\begin{array}{c} \frac{\sigma^{2}}{2}+\log\left(\sum_{k=1}^{K}e^{{\tilde{\mu }}_{k}}\right)=\overset{`}{\mu }+\frac{{\overset{`}{\sigma }}^{2}}{2}. \end{array}$$

Accordingly, we obtain

(A5) $$\overset{`}{\mu }=\log\left(\sum_{k=1}^{K}e^{{\tilde{\mu }}_{k}}\right)+\frac{1}{2}\left({\tilde{\sigma }}^{2}-{\overset{`}{\sigma }}^{2}\right).$$

By solving (A3) for ${\hat{\sigma }}^{2}$, (A3) can be written as

(A6) $$\begin{array}{cc} \; & \sum_{k=1}^{K}e^{2{\tilde{\mu }}_{k}}\left(e^{2{\tilde{\sigma }}^{2}}-e^{{\tilde{\sigma }}^{2}}\right)=\\ \; & e^{\left(2\log\left(\sum_{k=1}^{K}e^{{\tilde{\mu }}_{k}}\right)\right)}e^{\left({\tilde{\sigma }}^{2}-{\overset{`}{\sigma }}^{2}\right)}\left(e^{2{\overset{`}{\sigma }}^{2}}-e^{{\overset{`}{\sigma }}^{2}}\right). \end{array}$$

Consequentially, (A6) can be simplified to

(A7) $$\sum_{k=1}^{K}e^{2{\tilde{\mu }}_{k}}\left(e^{{\tilde{\sigma }}^{2}}-1\right)={\left(\sum_{k=1}^{K}e^{{\tilde{\mu }}_{k}}\right)}^{2}\left(e^{{\overset{`}{\sigma }}^{2}}-1\right).$$

Therefore, we can get

(A8) $$\overset{`}{\sigma }=\log\left(\frac{\sum_{k=1}^{K}e^{2{\tilde{\mu }}_{k}}}{{\left(\sum_{k=1}^{K}e^{{\tilde{\mu }}_{k}}\right)}^{2}}\left(e^{{\tilde{\sigma }}^{2}}-1\right)+1\right).$$

## References

[1] Zheng F., Kaiser T. (2016). Digital Signal Processing for RFID.

[2] El-Absi M., Alhaj Abbas A., Abuelhaija A., Zheng F., Solbach K., Kaiser T. (2018). High-Accuracy Indoor Localization Based on Chipless RFID Systems at THz Band. IEEE Access.

[3] Alhaj Abbas A., El-Absi M., Abualhijaa A., Solbach K., Kaiser T. (2019). Dielectric Resonator-Based Passive Chipless Tag With Angle-of-Arrival Sensing. IEEE Trans. Microw. Theory Tech..

[4] Li C., Mo L., Zhang D. (2019). Review on UHF RFID Localization Methods. IEEE J. Radio Freq. Identif..

[5] Miesen R., Ebelt R., Kirsch F., Schäfer T., Li G., Wang H., Vossiek M. (2011). Where is the Tag?. IEEE Microw. Mag..

[6] Guidi F., Decarli N., Dardari D., Mani F., D’Errico R. Passive Millimeter-Wave RFID Using Backscattered Signals. Proceedings of the IEEE Globecom Workshops (GC Wkshps).

[7] Pursula P., Donzelli F., Seppa H. (2011). Passive RFID at Millimeter Waves. IEEE Trans. Microw. Theory Tech..

[8] Patwari N., Hero A.O., Perkins M., Correal N.S., O’Dea R.J. (2003). Relative location estimation in wireless sensor networks. IEEE Trans. Signal Process..

[9] Patwari N., Ash J.N., Kyperountas S., Hero A.O., Moses R.L., Correal N.S. (2005). Locating the nodes: Cooperative localization in wireless sensor networks. IEEE Signal Process. Mag..

[10] Tomic S., Beko M., Dinis R. (2017). 3-D Target Localization in Wireless Sensor Networks Using RSS and AoA Measurements. IEEE Trans. Veh. Technol..

[11] Moragrega A., Closas P., Ibars C. (2015). Potential Game for Energy-Efficient RSS-Based Positioning in Wireless Sensor Networks. IEEE J. Sel. Areas Commun..

[12] So H.C., Lin L. (2011). Linear Least Squares Approach for Accurate Received Signal Strength Based Source Localization. IEEE Trans. Signal Process..

[13] Zhang S., Yang C., Jiang D., Kui X., Guo S., Zomaya A.Y., Wang J. (2019). Nothing Blocks Me: Precise and Real-Time LOS/NLOS Path Recognition in RFID Systems. IEEE Internet Things J..

[14] Gentile C., Alsindi N., Raulefs R., Teolis C. (2012). Geolocation Techniques: Principles and Applications.

[15] Werner M. (2014). Indoor Location-Based Services: Prerequisites and Foundations.

[16] Ciftler B.S., Kadri A., Güvenc I. (2017). IoT Localization for Bistatic Passive UHF RFID Systems with 3-D Radiation Pattern. IEEE Internet Things J..

[17] Zhao Y., Liu K., Ma Y., Gao Z., Zang Y., Teng J. (2017). Similarity Analysis-Based Indoor Localization Algorithm With Backscatter Information of Passive UHF RFID Tags. IEEE Sens. J..

[18] Zhang Z., Lu Z., Saakian V., Qin X., Chen Q., Zheng L.R. (2014). Item-Level Indoor Localization With Passive UHF RFID Based on Tag Interaction Analysis. IEEE Trans. Ind. Electron..

[19] Gao Z., Ma Y., Liu K., Miao X., Zhao Y. (2017). An Indoor Multi-Tag Cooperative Localization Algorithm Based on NMDS for RFID. IEEE Sens. J..

[20] Ciftler B.S., Kadri A., Guvenc I. Experimental performance evaluation of passive UHF RFID systems under interference. Proceedings of the IEEE International Conference on RFID Technology and Applications (RFID-TA).

[21] Zheng X., Liu H., Yang J., Chen Y., Martin R.P., Li X. (2014). A Study of Localization Accuracy Using Multiple Frequencies and Powers. IEEE Trans. Parallel Distrib. Syst..

[22] Ni L.M., Liu Y., Lau Y.C., Patil A.P. LANDMARC: Indoor location sensing using active RFID. Proceedings of the First IEEE International Conference on Pervasive Computing and Communications. (PerCom 2003).

[23] Zhao Y., Liu Y., Ni L.M. VIRE: Active RFID-based Localization Using Virtual Reference Elimination. Proceedings of the International Conference on Parallel Processing (ICPP 2007).

[24] Ni W., Xiao W., Toh Y.K., Tham C.K. Fingerprint-MDS based algorithm for indoor wireless localization. Proceedings of the 21st Annual IEEE International Symposium on Personal, Indoor and Mobile Radio Communications.

[25] Kumar V., Arablouei R., Jurdak R., Kusy B., Bergmann N.W. RSSI-based self-localization with perturbed anchor positions. Proceedings of the IEEE 28th Annual International Symposium on Personal, Indoor, and Mobile Radio Communications (PIMRC).

[26] Soro B., Lee C. (2019). Joint Time-Frequency RSSI Features for Convolutional Neural Network-Based Indoor Fingerprinting Localization. IEEE Access.

[27] Fu Y., Chen P., Yang S., Tang J. (2018). An Indoor Localization Algorithm Based on Continuous Feature Scaling and Outlier Deleting. IEEE Internet Things J..

[28] Hoang M.T., Yuen B., Dong X., Lu T., Westendorp R., Reddy K. (2019). Recurrent Neural Networks for Accurate RSSI Indoor Localization. IEEE Internet Things J..

[29] Li H., Qian Z., Tian C., Wang X. (2020). TILoc: Improving the robustness and accuracy for fingerprint-based indoor localization. IEEE Internet Things J..

[30] Guidi F., Decarli N., Dardari D., Mani F., D’Errico R. (2018). Millimeter-Wave Beamsteering for Passive RFID Tag Localization. IEEE J. Radio Freq. Identif..

[31] Vari M., Cassioli D. mmWaves RSSI indoor network localization. Proceedings of the IEEE International Conference on Communications Workshops (ICC).

[32] El-Absi M., Alhaj-Abbas A., Abuelhaija A., Solbach K., Kaiser T. (2020). Chipless RFID Infrastructure Based Self-Localization: Testbed Evaluation. IEEE Trans. Veh. Technol..

[33] Björnson E., Larsson E.G., Marzetta T.L. (2016). Massive MIMO: Ten myths and one critical question. IEEE Commun. Mag..

[34] Lu L., Li G.Y., Swindlehurst A.L., Ashikhmin A., Zhang R. (2014). An Overview of Massive MIMO: Benefits and Challenges. IEEE J. Sel. Top. Signal Process..

[35] Errington A.F.C., Daku B.L.F., Prugger A.F. (2010). Initial Position Estimation Using RFID Tags: A Least-Squares Approach. IEEE Trans. Instrum. Meas..

[36] Shirehjini A.A.N., Yassine A., Shirmohammadi S. (2012). An RFID-Based Position and Orientation Measurement System for Mobile Objects in Intelligent Environments. IEEE Trans. Instrum. Meas..

[37] Xu H., Wu M., Li P., Zhu F., Wang R. (2018). An RFID Indoor Positioning Algorithm Based on Support Vector Regression. Sensors.

[38] Xu H., Ding Y., Li P., Wang R., Li Y. (2017). An RFID Indoor Positioning Algorithm Based on Bayesian Probability and K-Nearest Neighbor. Sensors.

[39] Wang J., Ma Y., Zhao Y., Liu K. (2015). A Multipath Mitigation Localization Algorithm Based on MDS for Passive UHF RFID. IEEE Commun. Lett..

[40] Wang C., Shi Z., Wu F. (2017). Intelligent RFID Indoor Localization System Using a Gaussian Filtering Based Extreme Learning Machine. Symmetry.

[41] Koike-Akino T., Wang P., Pajovic M., Sun H., Orlik P.V. (2020). Fingerprinting-Based Indoor Localization with Commercial MMWave WiFi: A Deep Learning Approach. IEEE Access.

[42] Hu A., Lv T., Gao H., Zhang Z., Yang S. (2014). An ESPRIT-Based Approach for 2-D Localization of Incoherently Distributed Sources in Massive MIMO Systems. IEEE J. Sel. Top. Signal Process..

[43] Savic V., Larsson E.G. Fingerprinting-Based Positioning in Distributed Massive MIMO Systems. Proceedings of the IEEE 82nd Vehicular Technology Conference (VTC2015-Fall).

[44] Guidi F., Guerra A., Dardari D. (2016). Personal Mobile Radars with Millimeter-Wave Massive Arrays for Indoor Mapping. IEEE Trans. Mob. Comput..

[45] Guidi F., Guerra A., Dardari D. Millimeter-wave massive arrays for indoor SLAM. Proceedings of the IEEE International Conference on Communications Workshops (ICC).

[46] Garcia N., Wymeersch H., Larsson E.G., Haimovich A.M., Coulon M. (2017). Direct Localization for Massive MIMO. IEEE Trans. Signal Process..

[47] Vukmirović N., Janjić M., Djurić P., Erić M. (2018). Direct Localization for Massive MIMO. EURASIP J. Adv. Signal Process..

[48] Ni L.M., Zhang D., Souryal M.R. (2011). RFID-based localization and tracking technologies. IEEE Wirel. Commun..

[49] Kubina B., Schüßler M., Mandel C., Mehmood A., Jakoby R. (2013). Wireless high-temperature sensing with a chipless tag based on a dielectric resonator antenna. IEEE Sens..

[50] Abbas A., Abuelhaija A., Solbach K. Investigation of the transient EM scattering of a dielectric resonator. Proceedings of the 11th German Microwave Conference (GeMiC).

[51] El-Absi M., Abuelhaija A., Abbas A., Zheng F., Abbas A., Solbach K., Kaiser T. Chipless Tags Infrastructure based Localization in Indoor Environments. Proceedings of the German Microwave Conference.

[52] CST GmbH. https://www.cst-germany.de/.

[53] Abbas A.A., El-Absi M., Abuelhaijay A., Solbach K., Kaiser T. THz Passive RFID Tag Based on Dielectric Resonator Linear Array. Proceedings of the Second International Workshop on Mobile Terahertz Systems (IWMTS).

[54] Ramos A., Perret E., Rance O., Tedjini S., Lázaro A., Girbau D. (2016). Temporal Separation Detection for Chipless Depolarizing Frequency-Coded RFID. IEEE Trans. Microw. Theory Tech..

[55] Khaliel M., El-Awamry A., Megahed A.F., Kaiser T. (2017). A Novel Design Approach for Co/Cross-Polarizing Chipless RFID Tags of High Coding Capacity. IEEE J. Radio Freq. Identif..

[56] Bekkali A., Zou S., Kadri A., Crisp M., Penty R.V. (2015). Performance Analysis of Passive UHF RFID Systems Under Cascaded Fading Channels and Interference Effects. IEEE Trans. Wirel. Commun..

[57] Rappaport T. (2001). Wireless Communications: Principles and Practice.

[58] Azevedo J.A.R., Santos F.E.S. (2011). An Empirical Propagation Model for Forest Environments at Tree Trunk Level. IEEE Trans. Antennas Propag..

[59] El-Sayed H., Athanasiou G., Fischione C. Evaluation of localization methods in millimeter-wave wireless systems. Proceedings of the IEEE 19th International Workshop on Computer Aided Modeling and Design of Communication Links and Networks (CAMAD).

[60] Saab S.S., Nakad Z.S. (2011). A Standalone RFID Indoor Positioning System Using Passive Tags. IEEE Trans. Ind. Electron..

[61] Sahu P.K., Wu E.H.K., Sahoo J. (2013). DuRT: Dual RSSI Trend Based Localization for Wireless Sensor Networks. IEEE Sens. J..

[62] Hasani M., Lohan E.S., Sydänheimo L., Ukkonen L. Path-loss model of embroidered passive RFID tag on human body for indoor positioning applications. Proceedings of the IEEE RFID Technology and Applications Conference (RFID-TA).

[63] Piersanti S., Annoni L.A., Cassioli D. Millimeter waves channel measurements and path loss models. Proceedings of the IEEE International Conference on Communications (ICC).

[64] Maccartney G.R., Rappaport T.S., Sun S., Deng S. (2015). Indoor Office Wideband Millimeter-Wave Propagation Measurements and Channel Models at 28 and 73 GHz for Ultra-Dense 5G Wireless Networks. IEEE Access.

[65] Khalily M., Taheri S., Payami S., Ghoraishi M., Tafazolli R. (2018). Indoor wideband directional millimeter wave channel measurements and analysis at 26 GHz, 32 GHz, and 39 GHz. Trans. Emerg. Telecommun. Technol..

[66] Smulders P.F.M. (2009). Statistical Characterization of 60-GHz Indoor Radio Channels. IEEE Trans. Antennas Propag..

[67] Rappaport T.S., Xing Y., MacCartney G.R., Molisch A.F., Mellios E., Zhang J. (2017). Overview of Millimeter Wave Communications for Fifth-Generation (5G) Wireless Networks with a Focus on Propagation Models. IEEE Trans. Antennas Propag..

[68] Tarrío P., Bernardos A.M., Casar J. (2011). Weighted Least Squares Techniques for Improved Received Signal Strength Based Localization. Sensors.

[69] Johnson N., Kotz S., Balakrishnan N. (1995). Continuous Univariate Distributions.

[70] Mensing C., Plass S. Positioning Algorithms for Cellular Networks Using TDOA. Proceedings of the IEEE International Conference on Acoustics Speech and Signal Processing.

[71] Ouyang R.W., Wong A.K.S., Lea C.T., Zhang V.Y. Received Signal Strength-Based Wireless Localization via Semidefinite Programming. Proceedings of the IEEE Global Telecommunications Conference.

[72] Zhuang Y., Li Y., Lan H., Syed Z., El-Sheimy N. (2015). Wireless Access Point Localization Using Nonlinear Least Squares and Multi-Level Quality Control. IEEE Wirel. Commun. Lett..

[73] Tarrio P., Bernardos A.M., Besada J.A., Casar J.R. A new positioning technique for RSS-Based localization based on a weighted least squares estimator. Proceedings of the IEEE International Symposium on Wireless Communication Systems.

[74] Rao K.V.S., Nikitin P.V., Rao K.V.S., Nikitin P.V. (2006). Theory and measurement of backscattering from RFID tags. IEEE Antennas Propag. Mag..

[75] Manual W.I.U. (2003). Version 1.5.1. http://www.remcom.com/WirelessInSite/.

[76] El-Absi M., Galih S., Hoffmann M., El-Hadidy M., Kaiser T. (2016). Antenna Selection for Reliable MIMO-OFDM Interference Alignment Systems: Measurement-Based Evaluation. IEEE Trans. Veh. Technol..

[77] Fugen T., Maurer J., Kayser T., Wiesbeck W. (2006). Capability of 3-D Ray Tracing for Defining Parameter Sets for the Specification of Future Mobile Communications Systems. IEEE Trans. Antennas Propag..

[78] Rappaport T., Heath R., Daniels R., Murdock J. (2015). Millimeter Wave Wireless Communications.

[79] Zafari F., Gkelias A., Leung K.K. (2019). A Survey of Indoor Localization Systems and Technologies. IEEE Commun. Surv. Tutorials.

[80] Schwartz S.C., Yeh Y.S. (1982). On the distribution function and moments of power sums with log-normal components. Bell Syst. Tech. J..

